# Supplementary material for: Enzyme-assisted high throughput sequencing of an expanded genetic alphabet at single base resolution
Source: Nat Commun. 2024 May 14;15:4057. doi: 10.1038/s41467-024-48408-9 (PMC11094070; doi:10.1038/s41467-024-48408-9)
Supplement: Supplementary file 1 — Supplementary Information [file 41467_2024_48408_MOESM1_ESM.pdf]

Supporting information

Enzyme-Assisted High Throughput Sequencing of Expanded Genetic Alphabet at Single Base Resolution

Bang Wang[1,2], Kevin M. Bradley[3], Myong-Jung Kim[3], Roberto Laos[1], Cen Chen[1], Dietlind L. Gerloff[1], Luran Manfio[1], Zunyi Yang[1,3]*, Steven A. Benner[1,3]*

[1]Foundation for Applied Molecular Evolution, 13709 Progress Blvd, Alachua, FL, USA, 32615.

[2]Department of Chemistry, University of Florida, Gainesville, FL, USA, 32611.

[3]Firebird Biomolecular Sciences, LLC, Alachua, FL, USA, 32615

Zunyi Yang: zyang@ffame.org

Steven A. Benner: sbenner@ffame.org

Table of Contents

Materials or service 3

Machine or equipment 4

DNA sequence used in this study 5

Model of DNA sequencing analysis using restriction enzymes 7

DNA length extension for sanger sequencing 8

Primer extension 9

Effect of pH on PCR efficiency 10

Four-triphosphate qPCR analysis 10-fold diluted Nat and ZZ template amplification 11

Four-triphosphate qPCR analysis 10-fold diluted ZP-1 and ZP-2 template amplification 12

Five-triphosphate qPCR analysis 10-fold diluted ZP-1 and ZP-2 template amplification .13

Five-triphosphate PCR 14

Sanger sequencing chromatograms for model DNA ZP-1 15

Sanger sequencing chromatograms for model DNA ZP-2 16

Evaluation of the fidelity of 6-triphosphate PCR 17

Evaluation of the fidelity of different modified dPTPs in 6-triphosphate PCR 18

Z-ran template assess the fidelity of different modified dPTPs in 6-triphosphate PCR 19

6M Taq DNA polymerase 20

Synthesis of 7-functionalized-dPTP derivatives 26

HPLC and mass spectrometry of ZZ, ZP-1, ZP-2 sequence 29

Analysis of Z-Ran sequence by HPLC 32

Analysis of P-Ran sequence by HPLC 33

NMR spectrum of 2a 34

HRMS spectrum of 2a 36

NMR spectrum of 2b 36

HRMS spectrum of 2b 37

NMR spectrum of 3a 38

HRMS spectrum of 3a 39

NMR spectrum of 3b 40

HRMS spectrum of 3b 41

Supplementary Note 1: The C# code 42

References 50

Materials or service.

Name	Source/Company	Catalog
Standard DNA	Integrated DNA technology	--
AEGIS DNA	Firebird Biomolecular Sciences	--
AluI	NEB (New England BioLabs)	R0137S
PspOMI	NEB (New England BioLabs)	R0653S
SYBR-Gold	ThermoFisher Scientific	S11494
Sanger sequencing and NGS	Genewiz	--
Enzymatic Methyl-Seq kit (APOBEC)	NEB (New England BioLabs)	E7120S
AMPure XP Reagent	BeckMan	A63880
TaKaRa Taq^{TM} Hot start polymerase	TaKaRa	R007A
d(A, T, C, G) TP	TaKaRa	R007A
10X PCR Buffer (pH8.9)	TaKaRa	R007A
Formamide	Sigma	75-12-7
dZTP	Firebird Biomolecular Sciences	dZTP-101
dPTP	Firebird Biomolecular Sciences	dPTP-201
GeneJET gel extraction kit	ThermoFisher Scientific	K0692
T4 Polynucleotide Kinase	NEB (New England BioLabs)	M0201S
[r-^{32}P] ATP	PekinElmer	BLU002Z250UC
QIAquick Nucleotide Removal Kit	QIAGEN	28304
EvaGreen® Dye, 20X	Biotium	#31000-T
C18 cartridges	Sep-Pak	WAT020515
KOD exo^{-} DNA Polymerase	Sekisui	KODND-109B
KlenTaq1 DNA Polymerase	AB Bioscience LLC	1001
Phire hot start II DNA Polymerase	Thermo Scientific	F122S
Phusion TM DNA Polymerase	Thermo Scientific	F530S
Go Taq DNA Polymerase	Promega	PR-M8291
One Taq DNA Polymerase	New England Biolabs, Inc	NC0108355
LongAmp Taq DNA Polymerase	New England Biolabs, Inc	50-995-156
Q5 High-Fidelity DNA Polymerase	New England Biolabs, Inc	50-591-065
Sulfolobus DNA Polymerase	New England Biolabs, Inc	50-436-5
Vent exo^{-} DNA Polymerase	New England Biolabs, Inc	50-811-673
HiFi KAPA DNA Polymerase	Roche Diagnostics	50-196-5310
Trizma® hydrochloride BioXtra	Sigma Aldrich	T6666
Trizma® base BioXtra	Sigma Aldrich	T6791
UltraPure™ Glycerol	Invitrogen	15514-011
LB Broth	Apex BioResearch Products	11-120
Agar, Powdered bacteriological grade	Apex BioResearch Products	20-273
Ni-NTA Agarose	Invitrogen	R901-15
BugBuster® Master Mix	Novagen	71456-4
pJExpress414 vector	DNA 2.0	--
Amicon™Ultra-15, 50,000 MWCO	MilliporeSigma™	UFC903008
Tween® 20	Sigma Aldrich	P9416
Igepal	Sigma Aldrich	I3021
Pierce™ BCA Protein Assay Kit	Thermo Scientific™	23228
AEGIS-DNA Mass spectral analysis	Novatia	ESI
Nucleoside Mass spectral analysis	University of Florida	ESI
Triphosphate Mass spectral analysis	Novatia	ESI

Machine or equipment.

Name	Source/Company	Model/Catalog No.
Typhoon Imaging System	Cytiva	Amersham TYPHOON
PCR machine	Bio-Rad	T100
qPCR	Bio-Rad	CFX Opus 96
Autoradiography	Bio-Rad	PMI
Nanodrop One	Thermo Scientific™	13-400-519
Electronic pH meter	Accumet Basic	AB15
DNA synthesizer	Bioautomation	Mermade 12
HPLC	Thermo Fisher Scientific Inc	Ultimate 3000
Centrifuge	Eppendorf	5804
Centrifuge	Thermo Fisher Scientific Inc	Legend Micro 21
NMR	Varian Mercury	M300-mercury300

Supplementary Table 1 | DNA sequence used in this study.

Source	Name	Sequence (5'-3')
IDT	F-Primer22	TAAGATGAGAGTTGAGGAGAGT
IDT	R-Primer24	TCCACTATCTACTTACACTACTAT
IDT	Nat-Temp	TAAGATGAGAGTTGAGGAGAGTTATCCAAGCTATAGGGCCCTTCAGTATAGTAGTGTAAGTAGAT<br>AGTGGA
Firebird	ZZ	TAAGATGAGAGTTGAGGAGAGTTATCCAAGZTATAGGGCZZTTCAGTATAGTAGTGTAAGTAGAT<br>AGTGGA
Firebird	ZP- 1	TAAGATGAGAGTTGAGGAGAGTTACGTGZACGCPTPGTCAZCACAGTATAGTAGTGTAAGTAGAT<br>AGTGGA
Firebird	ZP- 2	TAAGATGAGAGTTGAGGAGAGTTATCAPCGTAGCAZPCTTPTZATGTATAGTAGTGTAAGTAGAT<br>AGTGGA
IDT	C-Ran	TAAGATGAGAGTTGAGGAGAGTTATNNNCNNNGTATAGTAGTGTAAGTAGATAGTGGA
Firebird	Z-Ran	TAAGATGAGAGTTGAGGAGAGTTATNNNZNNNGTATAGTAGTGTAAGTAGATAGTGGA
Firebird	P-Ran	TAAGATGAGAGTTGAGGAGAGTTATNNNPNNNGTATAGTAGTGTAAGTAGATAGTGGA
	A1-200 (200bp)	ATGATGAAGCCTGAAGTTCCAGATTCGCAGCCTTTGTGAACCAACACCCATCAGTGTCAACAGCT<br>GCGTGA<br>TTTTCTTGTACAGGTCCCGCTCTTTATTCTGCGCTGATCATGCATTTGTATGCCATGTGGACGAC<br>CAGAGC<br>CAGACCCGCAGATGCTGGACGACAGATCCTCACCTAAGATGAGAGTTGAGGAGAGTTA
	A1-100	ATGATGAAGCCTGAAGTTCCAGATTCCCATCAGTGATTCTGCGCTGATCATGCTGTATGCCATGT<br>GCATCCTCACCTAAGATGAGAGTTGAGGAGAGTTA
	A1-primer	ATGATGAAGCCTGAAGTTCC
	B1-100 (100bp)	GGTGTTAGTCTTGCTGTTGCTGTGACCTCCGAGAAGTGGGTGGCTTATCCAATCGGTAGTAGCGG<br>TGACCTTCTGCTCCACTATCTACTTACACTACTAT
IDT	B1-primer	GGTGTTAGTCTTGCTGTTGC
	A1-Bod-1	ACACTCTTTCCCTACACGACGCTCTTCCGATCTGAGTCCATATGATGAAGCCTGAAGTTCCA
	A1-Bod-2	ACACTCTTTCCCTACACGACGCTCTTCCGATCTTAGTCGAGATGATGAAGCCTGAAGTTCCA
	A1-Bod-3	ACACTCTTTCCCTACACGACGCTCTTCCGATCTCCGATACGATGATGAAGCCTGAAGTTCCA
	A1-Bod-4	ACACTCTTTCCCTACACGACGCTCTTCCGATCTTTGCACTGATGATGAAGCCTGAAGTTCCA
IDT	A1-Bod-5	ACACTCTTTCCCTACACGACGCTCTTCCGATCTGAAGATTCATGATGAAGCCTGAAGTTCCA
	B1-Bod-1	GACTGGAGTTCAGACGTGTGCTCTTCCGATCTCTTCACGAGGTGTTAGTCTTGCTGTTGCT
	B1-Bod-2	GACTGGAGTTCAGACGTGTGCTCTTCCGATCTTGAAGCTCGGTGTTAGTCTTGCTGTTGCT
	B1-Bod-3	GACTGGAGTTCAGACGTGTGCTCTTCCGATCTGAGATGCTGGTGTTAGTCTTGCTGTTGCT
	B1-Bod-4	GACTGGAGTTCAGACGTGTGCTCTTCCGATCTGCAGTATCGGTGTTAGTCTTGCTGTTGCT
IDT	B1-Bod-5	GACTGGAGTTCAGACGTGTGCTCTTCCGATCTAACTCGTCGGTGTTAGTCTTGCTGTTGCT
IDT	Seq-FP	ACACTCTTTCCCTACACGACGCT
IDT	Seq-RP	GACTGGAGTTCAGACGTGTGCT

Note: The red labeled sequences represent barcodes used in Next Generation Sequencing.

Supplementary Table 2 | DNA sequence and primers used in this study.

Source	Name	Sequence (5’-3’)
IDT	F-primer-22-FAM	/56-FAM/TAAGATGAGAGTTGAGGAGAGT
IDT	R-primer-24-spc-cy5	/5Cy5/TTTTTTTTGTTTGTTTTTTTTTTTTTT/isp18/TCCACTATCTACTTACACTACTAT
IDT	C-100	ACATCATTTACCTTCACTTACTAGACTCCTGGCGTACTGGCCTACTAGTGCCTTCCCATCGGACACGCATGGCGTACTTAAAATAAAAATTAAAAAAAAT
IDT	D-100	ATCTTACCTAACCCTTAACCTCGTACGGCCTAGCGTACCGGTTACGGGCTCCCATGCCCTTTCTGCCGAAATGGGTTTTATTATTTATTTATATTATTAT
IDT	C-bod-N1	ACACTCTTTCCCTACACGACGCTCTTCCGATCTATTCGGCATTCGGCATTCGGACATCATTTACCTTCACTTACTA
	C-bod-N2	ACACTCTTTCCCTACACGACGCTCTTCCGATCTATTCGGCATTCGGTCATCCGACATCATTTACCTTCACTTACTA
	C-bod-N3	ACACTCTTTCCCTACACGACGCTCTTCCGATCTATTCGGCATTCGGGACTGATACATCATTTACCTTCACTTACTA
	C-bod-N4	ACACTCTTTCCCTACACGACGCTCTTCCGATCTATTCGGCATTCGGTACTGGTACATCATTTACCTTCACTTACTA
	C-bod-N5	ACACTCTTTCCCTACACGACGCTCTTCCGATCTATTCGGCATTCGGCGTCTATACATCATTTACCTTCACTTACTA
IDT	D-bod-N1	GACTGGAGTTCAGACGTGTGCTCTTCCGATCTAGCGCTTAGTGCTTAGGCTTATCTTACCTAACCCTTAACCT
	D-bod-N2	GACTGGAGTTCAGACGTGTGCTCTTCCGATCTAGCGCTTAGTGCCTCAGTGCATCTTACCTAACCCTTAACCT
	D-bod-N3	GACTGGAGTTCAGACGTGTGCTCTTCCGATCTAGCGCTTAGTGCAGTGCTAAATCTTACCTAACCCTTAACCT
	D-bod-N4	GACTGGAGTTCAGACGTGTGCTCTTCCGATCTAGCGCTTAGTGCTCAAGTGCATCTTACCTAACCCTTAACCT
	D-bod-N5	GACTGGAGTTCAGACGTGTGCTCTTCCGATCTAGCGCTTAGTGCCGCATATCATCTTACCTAACCCTTAACCT

Note: The red labeled sequences represent barcodes used in Next Generation Sequencing.

Model of DNA sequencing analysis using restriction enzymes.

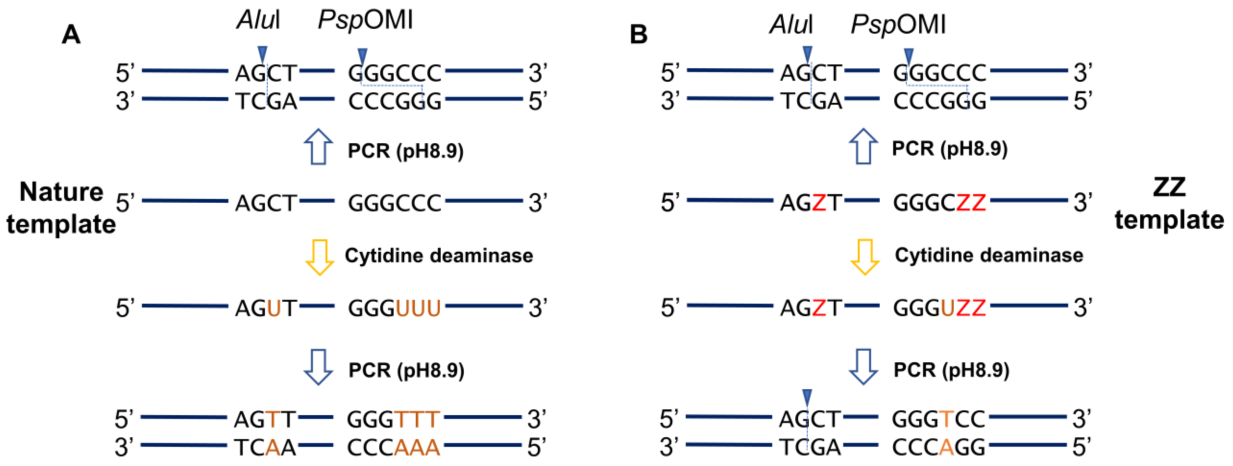

Supplementary Figure 1 | Schematic of the DNA sequencing analysis. (A) Workflow for sequencing a "Nat" template (natural DNA sequence with only A, T, G, and C as building blocks), which treated or not treated with the APOBEC deaminase enzyme prior to PCR amplification and restriction enzyme digestion. (B) Workflow for sequencing a ZZ template, which is treated or not treated with the APOBEC deaminase enzyme prior to PCR and restriction enzyme digestion.

DNA length extension

A

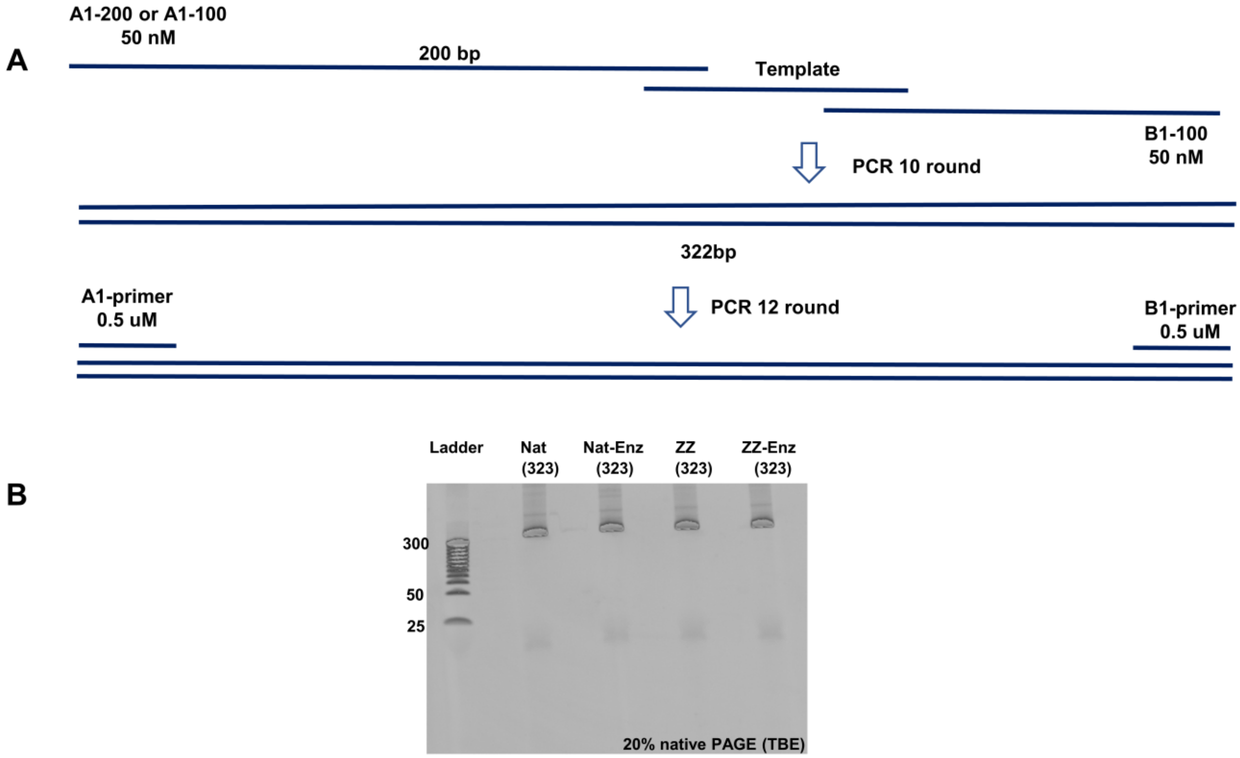

Supplementary Figure 2 | Analysis of DNA length extension. (A) Schematic of process to extend the length of the DNA prior to sequencing. Note: A1-200 primer using for Sanger Sequencing, A1-100 primer using for NGS. (B) Analysis of the PCR product obtained after length extension using 20% Native PAGE (TBE). The gel was stained with SYBR-Gold and imaged using a Typhoon scanner with the Cy2 channel. The resulting image shows the migration pattern of the extended DNA product on the gel, which can be used to confirm successful extension and estimate the length of the DNA fragments. Uncropped gel was provided in Source data file.

Primer extension

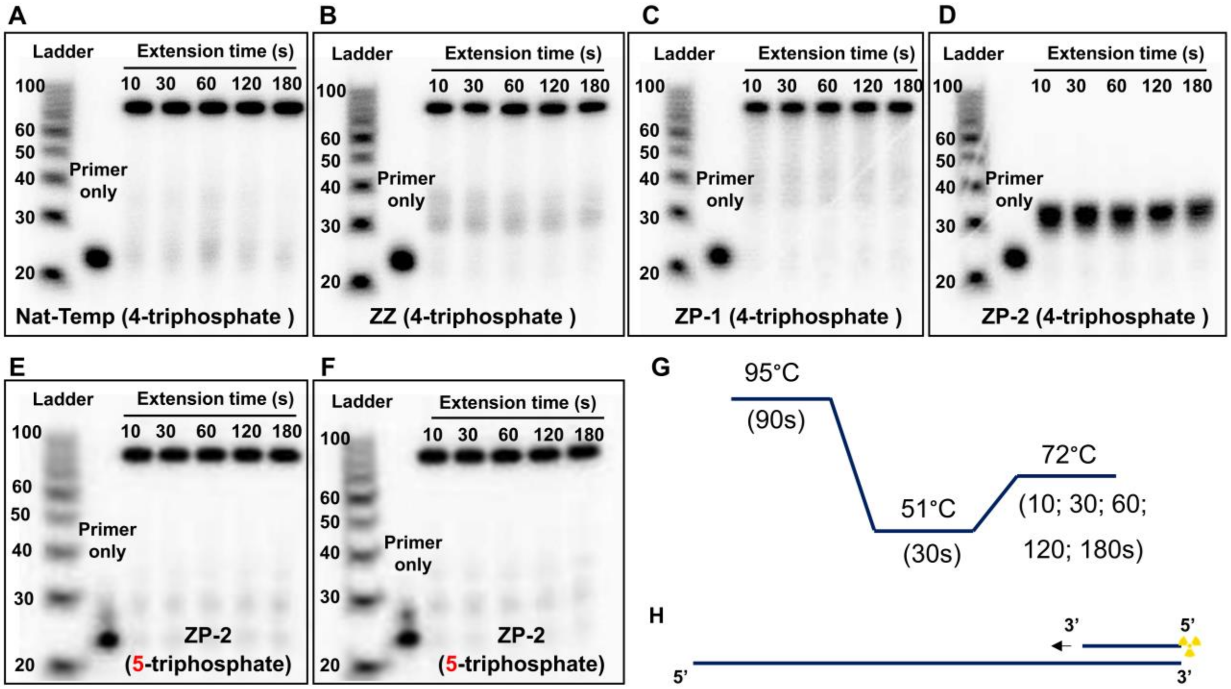

Supplementary Figure 3 | Analysis of primer extension products using TBE-Urea PAGE (15%). (A) Primer extension reaction using natural template and four standard nucleoside triphosphates. d(ATCG)TP (0.2 mM each). (B) Four-triphosphate primer extension reactions using the ZZ sequence template. (C) Four-triphosphate primer extension reactions using the ZP-1 template. (D) Four-triphosphate primer extension reactions using the ZP-2 template. (E) 5-triphosphate primer extension reaction for ZP-2 template. d (A. T, C, G) TP (0.2 mM); dZTP (0.1 mM). (F) Five-triphosphate primer extension reactions using ZP-2 template. d(ATCG)TP (0.2 mM) with dZTP (0.05 mM). (G) Temperature cycle for primer extension reactions. (H) Schematic of primer extension reaction using a 5'-^{32}P labeled primer, which can be used to visualize the extended products by autoradiography. Note: Uncropped gel was provided in Source data file.

Effect of pH on PCR efficiency

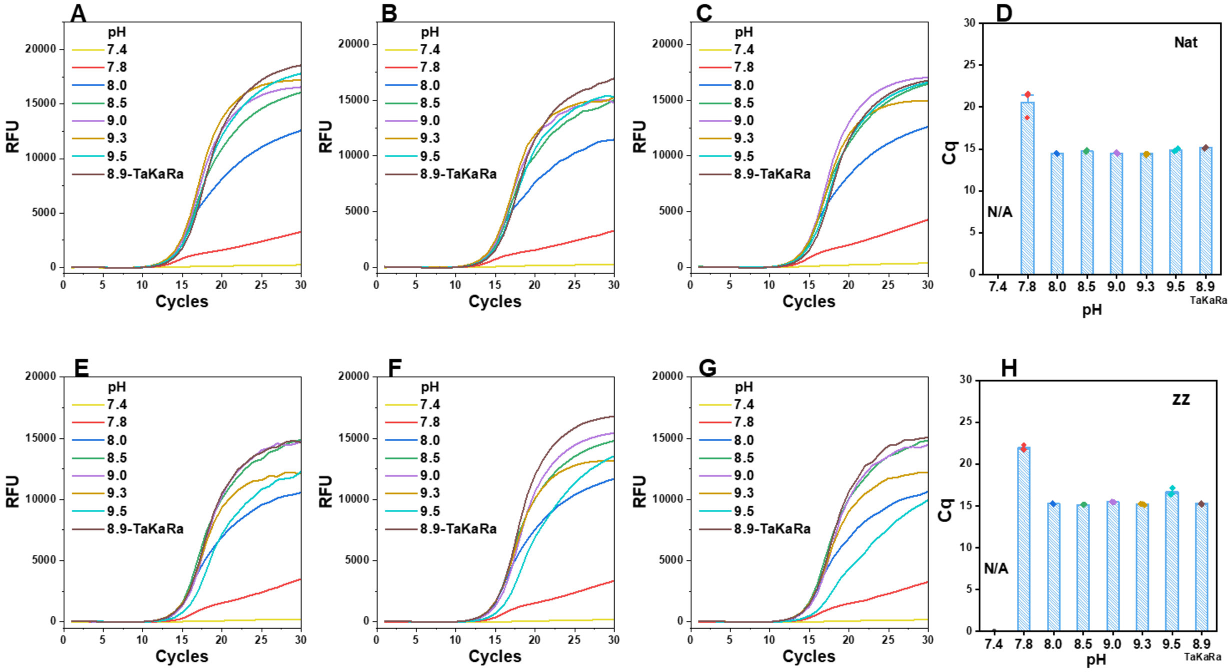

Supplementary Figure 4 | Effect of pH on the efficiency of 4-triphosphate qPCR analysis using Natural and ZZ templates. (A, B, C) qPCR curves obtained using the natural template at different pH values. A, B, and C represent three independent replicates. (D) Quantification cycle (C_q) values of qPCR for the natural template at different pH values. Error bars represent the standard deviation of three independent experiments (n=3; mean ± SD). (E, F, G) qPCR curves of the ZZ template at different pH values. E, F, and G represent three independent replicates. (H) Cq values of qPCR for the ZZ template at different pH values. Error bars represent the standard deviation of three independent experiments (n=3; mean ± SD). Note: Buffers with pH values of 7.4, 7.8, 8.0, 8.5, 9.0, and 9.3 were prepared in-house. The pH 8.9 buffer was provided by TaKaRa. Fluorescent signal generated by Evagreen in quantitative PCR (qPCR) analysis. Note: All statistical source data are available in the "Source Data" file.

Four-triphosphate qPCR analysis 10-fold diluted Nat and ZZ template amplification

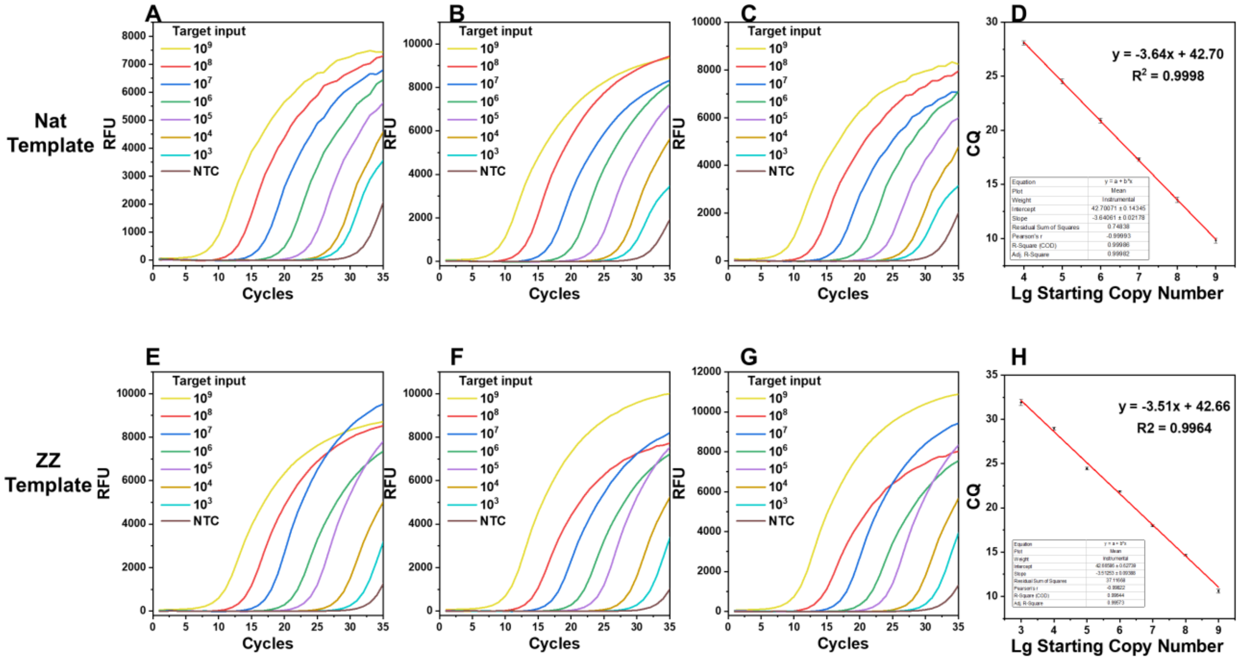

Supplementary Figure 5 | Amplification plot and standard curve of 10-fold diluted Nat and ZZ templates using 4-triphosphate qPCR. (A-C) qPCR amplification plots of Nat template DNA at serial dilutions, with A, B, and C representing three parallel experiments. (D) Standard curve generated from mean C_q values of Nat template. y = -3.64x + 42.70. R^2 = 0.9998. Error bars represent the standard deviation of three independent experiments (n=3; mean ± SD). (E-G) qPCR amplification plots of serially diluted ZZ template DNA, with E, F, and G representing three parallel experiments. (H) Standard curve (y = -3.51x + 42.66. R^2 = 0.9964) generated from mean C_q values of Nat template. Error bars represent the standard deviation of three independent experiments (n=3; mean ± SD). Eva Green was used to generate all curve signals. Note: All statistical source data are available in the "Source Data" file.

Four-triphosphate qPCR analysis 10-fold diluted ZP-1 and ZP-2 template amplification

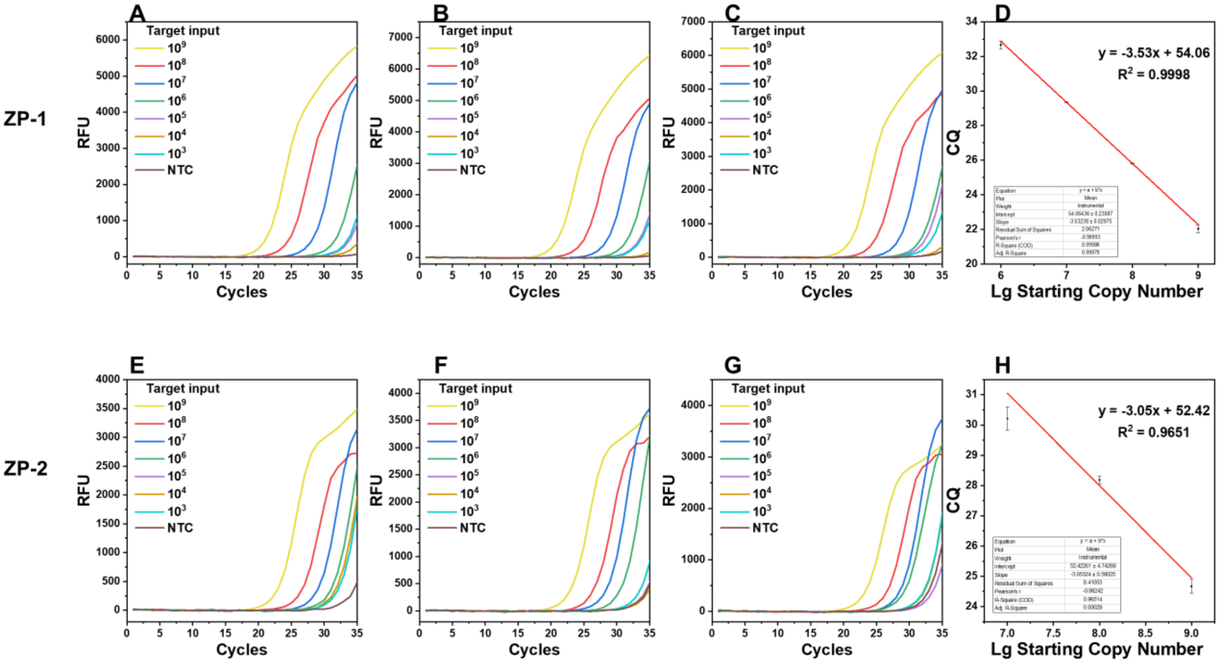

Supplementary Figure 6 | Amplification plot and standard curve of 10-fold diluted ZP-1 and ZP-2 templates using 4-triphosphate qPCR. (A-C) qPCR amplification plots of ZP-1 template DNA at serial dilutions, with A, B, and C representing three parallel experiments. (D) Standard curve (y = -3.53x + 54.06. R^2 = 0.9998) generated from mean C_q values of Nat template. Error bars represent the standard deviation of three independent experiments (n=3; mean ± SD). (E-G) qPCR amplification plots of serially diluted ZP-2 template DNA, with E, F, and G representing three parallel experiments. (H) Standard curve (y = -3.05x + 52.42. R^2 = 0.9651) generated from mean C_q values of Nat template. Error bars represent the standard deviation of three independent experiments (n=3; mean ± SD). Eva Green was used to generate all curve signals. Note: All statistical source data are available in the "Source Data" file.

Five-triphosphate qPCR analysis 10-fold diluted ZP-1 and ZP-2 template amplification

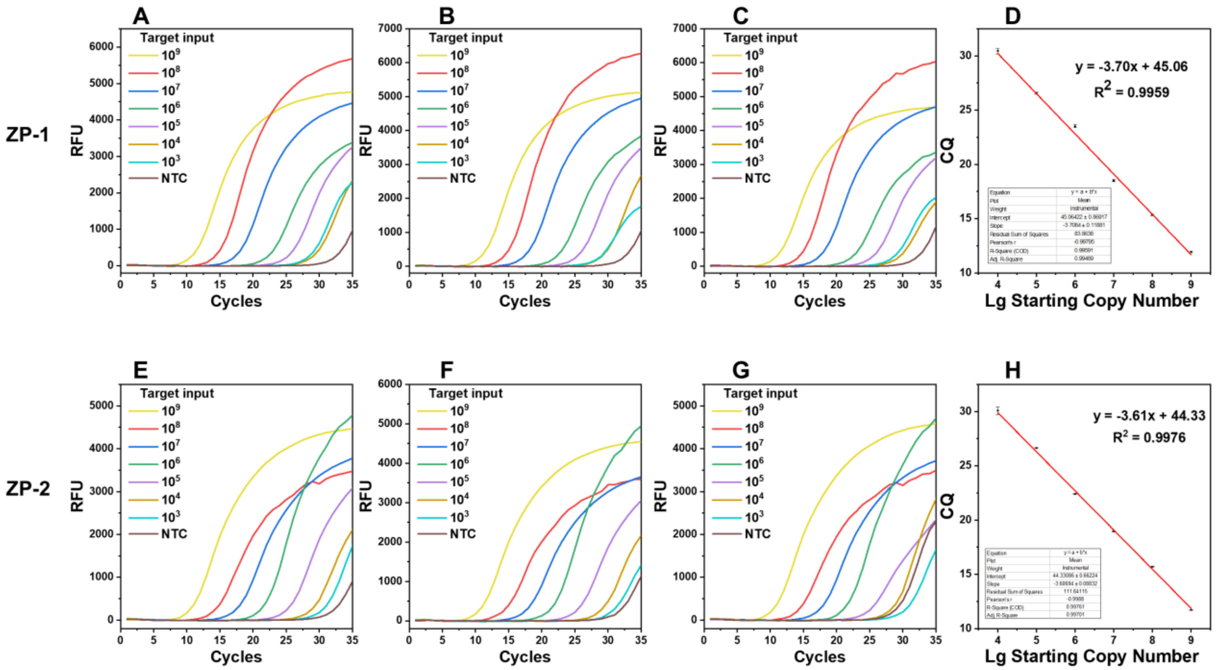

Supplementary Figure 7 | Amplification plot and standard curve of 10-fold diluted ZP-1 and ZP-2 templates using 5-triphosphate qPCR. (A-C) Five-triphosphate (fur standard plus dZTP) qPCR amplification plots of ZP-1 template DNA at serial dilutions, with A, B, and C representing three parallel experiments. (D) Standard curve (y = -3.70x + 45.06. R^2 = 0.9959) generated from mean C_q values of Nat template. Error bars represent the standard deviation of three independent experiments (n=3; mean ± SD). (E-G) 5-triphosphate qPCR amplification plots of serially diluted ZP-2 template DNA, with E, F, and G representing three parallel experiments. (H) Standard curve (y = -3.61x + 44.33. R^2 = 0.9976) generated from mean C_q values of Nat template. Error bars represent the standard deviation of three independent experiments (n=3; mean ± SD). Eva Green was used to generate all curve signals. Note: All statistical source data are available in the "Source Data" file.

.

Five-triphosphate PCR

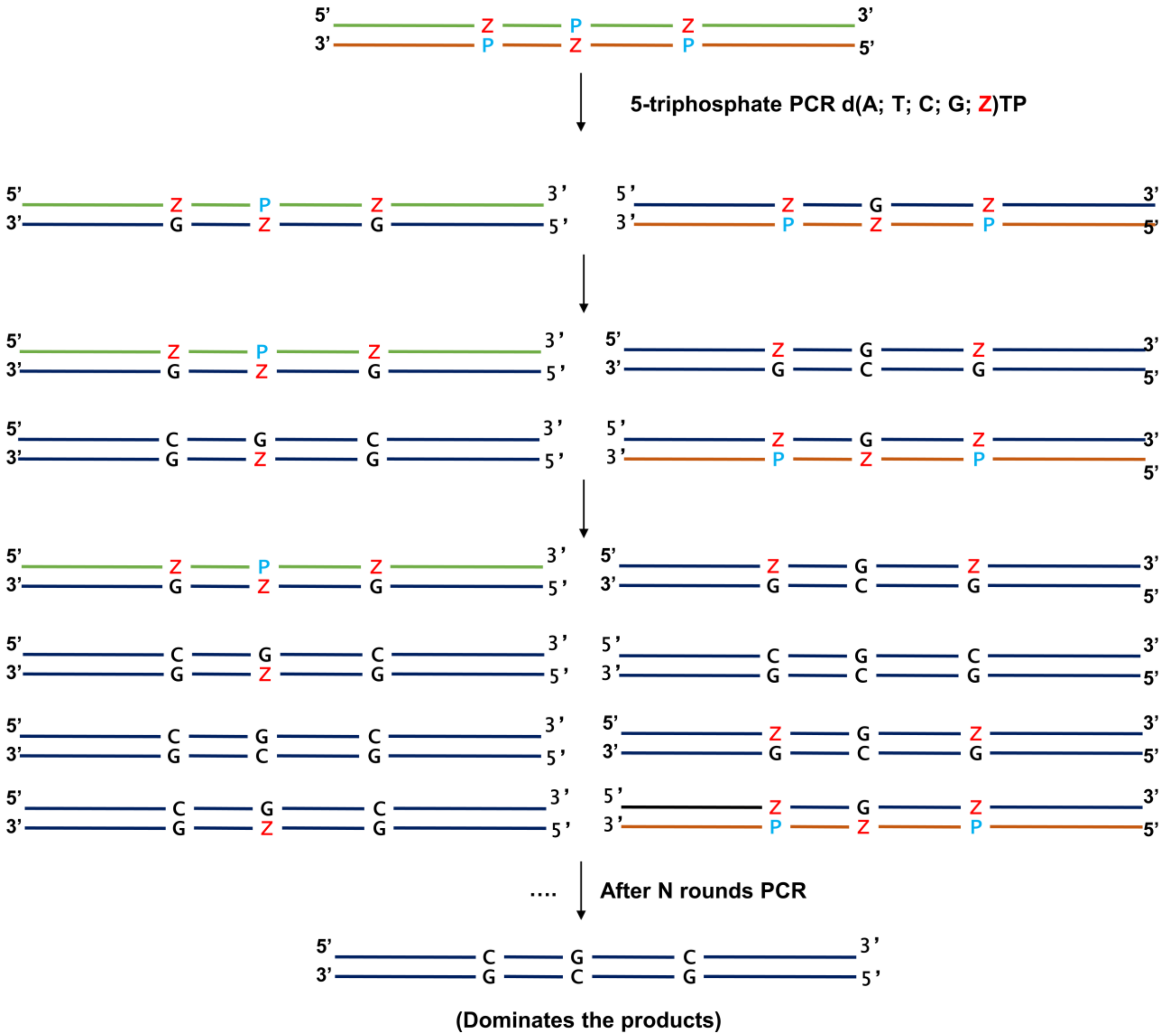

Supplementary Figure 8 | Schematic of 5-triphosphate PCR replace the Z: P pair to C: G pair with highly fidelity.

Sanger sequencing chromatograms for model DNA ZP-1

ZP-1	TAAGATGAGAGTTGAGGAGAGT*TACGTGZACGCPTPGTCAZCACAGT*ATAGTAGTGTAAGTAGATAGTGGA

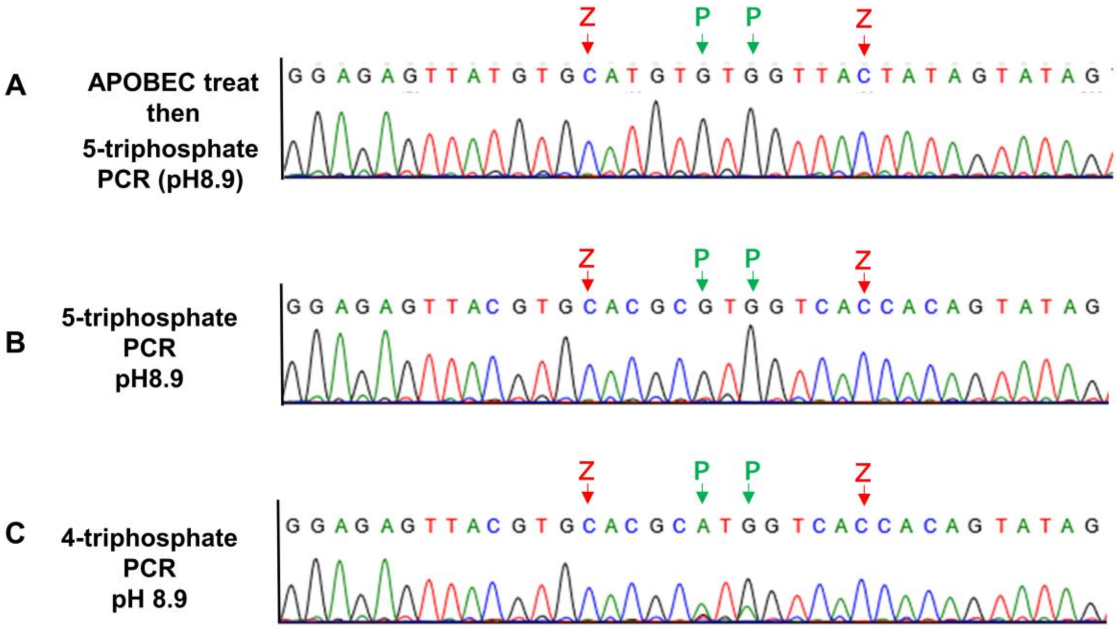

Supplementary Figure 9 | Sanger sequencing chromatograms for model DNA (ZP-1) containing both Z and P under different PCR conditions. (A) The ZP-1 sequence treated with the APOBEC deaminase enzyme to deaminate cytosines to uracils, followed by PCR with 5-triphosphate d(A, T, C, G, Z)TP and TaKaRa Taq HS polymerase (pH 8.9). (B) ZP-1 DNA sample subjected to 5-triphosphate PCR with TaKaRa Taq HS polymerase (pH 8.9). (C) ZP-1 DNA sample subjected to 4-triphosphate PCR with TaKaRa Taq HS polymerase (pH 8.9).

Sanger sequencing chromatograms for model DNA ZP-2

ZP-2	TAAGATGAGAGTTGAGGAGAGT*TATCAPCGTAGCAZPCTTPTZATGT*ATAGTAGTGTAAGTAGATAGTGGA

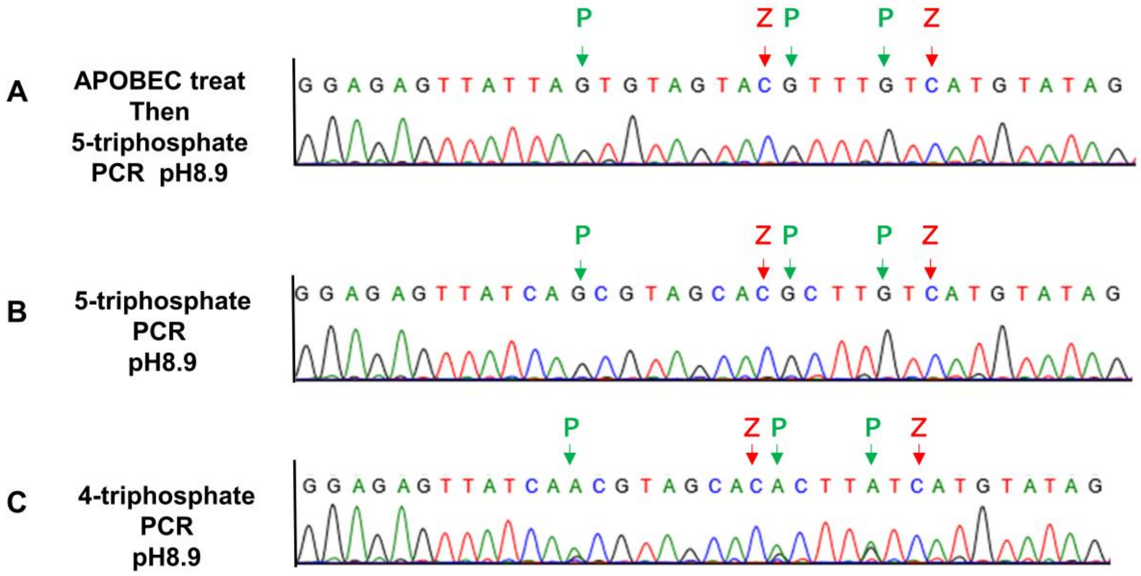

Supplementary Figure 10 | Sanger sequencing chromatograms for model DNA (ZP-2) containing both Z and P under different PCR conditions. (A) ZP-2 sequence treated with APOBEC deaminase enzyme to deaminate cytosines to uracils, followed by PCR with 5-triphosphate d(A, T, C, G, Z)TP and TaKaRa Taq HS polymerase (pH 8.9). (B) ZP-2 DNA sample subjected to 5-triphosphate PCR with TaKaRa Taq HS polymerase (pH 8.9). (C) ZP-2 DNA sample subjected to 4-triphosphate PCR with TaKaRa Taq HS polymerase (pH 8.9).

Evaluation of the fidelity of 6-triphosphate PCR.

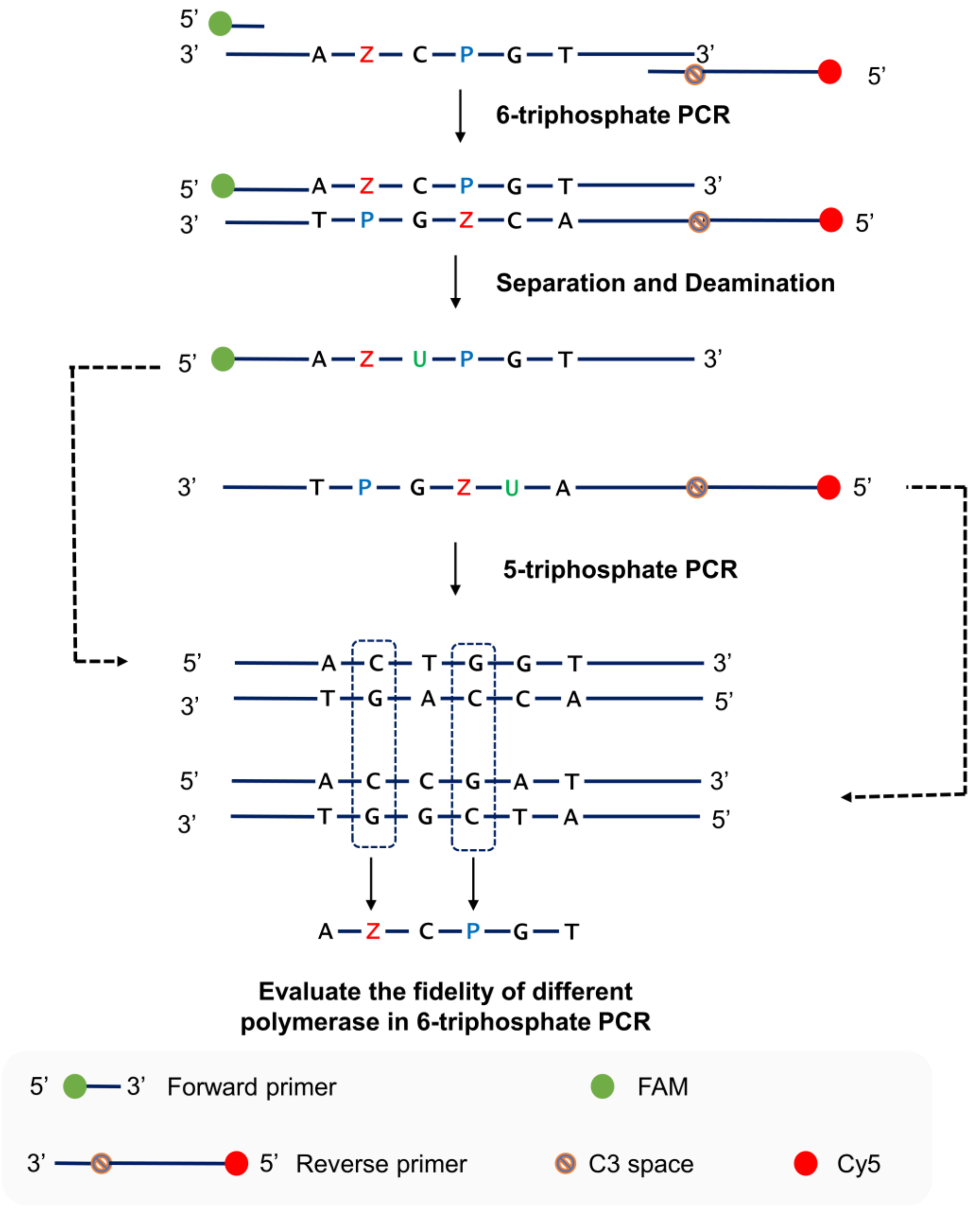

Supplementary Figure 11 | Workflow of Evaluation of the fidelity of 6-triphosphate PCR under various condition including: pH value, the concentration of dPTP, and various DNA polymerases.

Evaluation of the fidelity of different modified dPTPs in 6-triphosphate PCR.

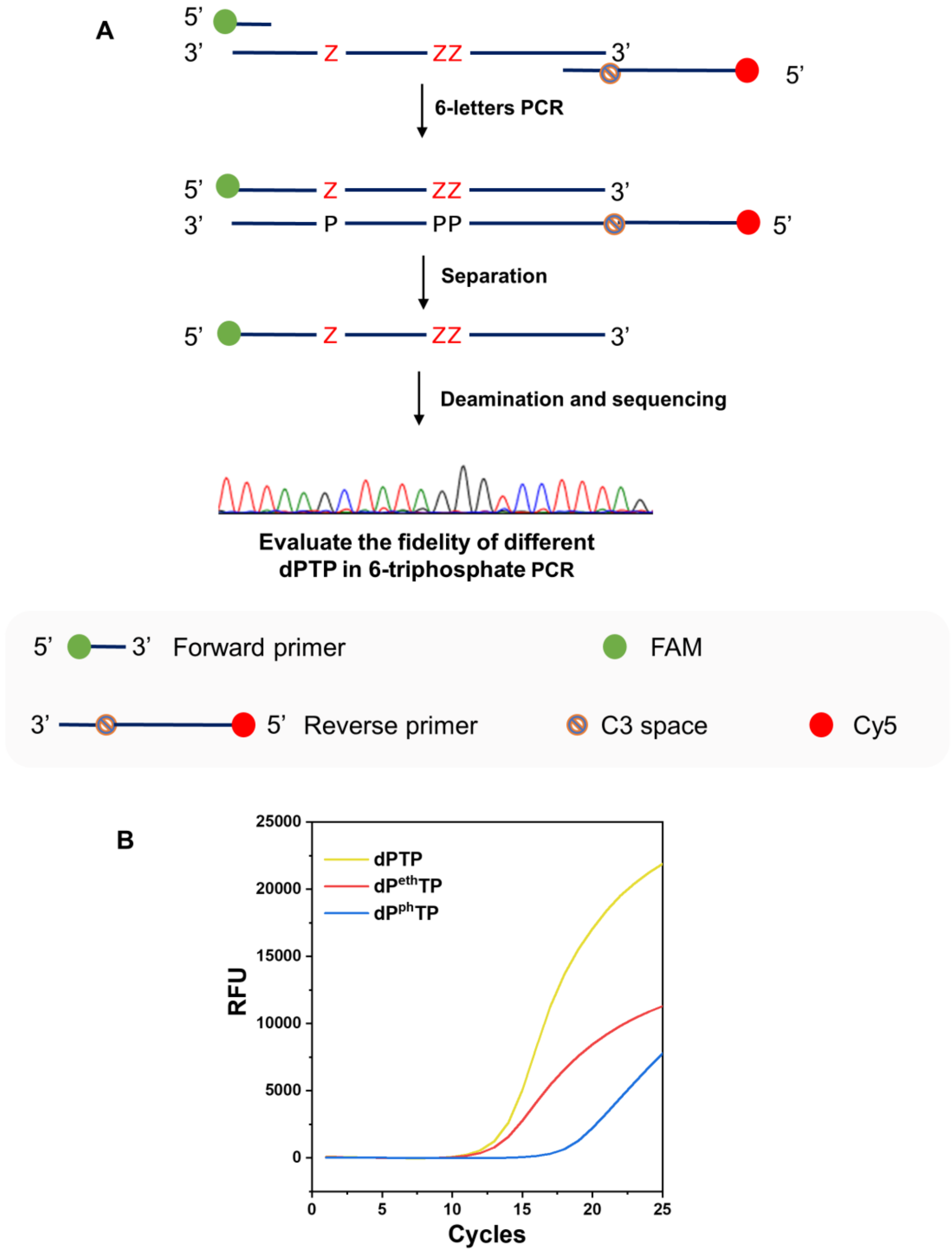

Supplementary Figure 12 | (A) Workflow of Evaluation of the fidelity of different modified dPTPs in 6-triphosphate PCR by using ZZ template. (B) qPCR monitoring of the 6-triphosphate PCR process with different modified dPTPs and unlabeled primers (Fluorescence Originating from Eva Green). Note: All statistical source data are available in the "Source Data" file.

Z-ran template assessed the fidelity of different modified dPTPs in 6-triphosphate PCR

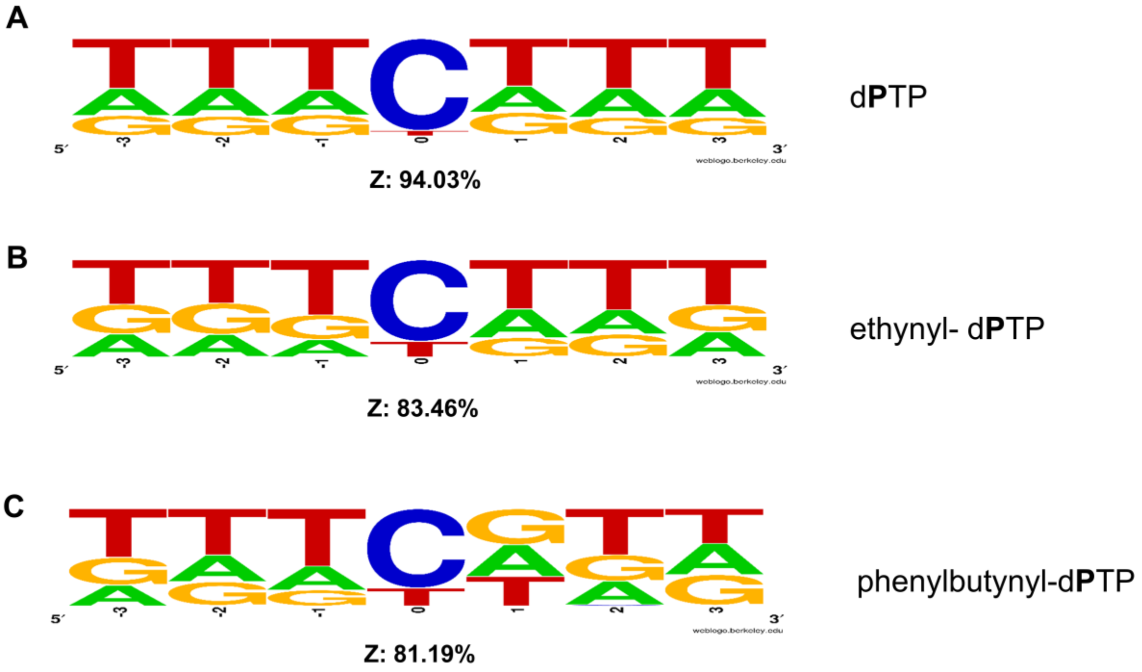

Supplementary Figure 13 | Sequence logos of ESEGA sequencing of 6-triphosphate PCR product from Z-Ran template. (A). The 6-triphosphates contain dATP, dTTP, dCTP, dGTP, dZTP, dPTP. (B). The 6-triphosphates contain dATP, dTTP, dCTP, dGTP, dZTP, $dP^{eth}TP$. (C). The 6-triphosphates contain dATP, dTTP, dCTP, dGTP, dZTP, $dP^{ph}TP$. Note: All statistical source data are available in the "Source Data" file.

6M Taq DNA polymerase variant

This is a Taq polymerase variant with a total of six mutations. The Taq polymerase has been truncated (the first 280 amino acids have been deleted). On the N-terminus the DNA binding domain from Sulfolobulos solfactarius (Sso7d)

Sso7d-*Taq*(Δ1-280) [R587Q; E626K; I707L; E708K; A743H; E832G]

Mutation positions numbering is with respect to DPO1_THEAQ:

[R587Q; E626K; I707L; E708K; A743H; E832G]

```
1   MASRGSHHHH HHGAGDLMAT VKFKYKGEEK EVDISKIKKV WRVGKMISFT YDEGGGKTGR
61  GAVSEKDAPK ELLQMLEKQK KGSLLHEFGL LESPKALEEA PWPPPEGAFV GFVLSRKEPM
121 WADLLALAAA RGGRVHRAPE PYKALRDLKE ARGLLAKDLS VLALREGLGL PPGDDPMLLA
181 YLLDPSNTTP EGVARRYGGE WTEEAGERAA LSERLFANLW GRLEGEERLL WLYREVERPL
241 SAVLAHMEAT GVRLDVAYLR ALSLEVAEEI ARLEAEVFRL AGHPFNLNSR DQLERVLFDE
301 LGLPAIGKTE KTGKRSTSAA VLEALREAHP IVEKILQYRE LTKLKSTYID PLPDLIHPRT
361 GRLHTRFNQT ATATGRLSSS DPNLQNIPVQ TPLGQRIRRA FIAEEGWLLV ALDYSQIELR
421 VLAHLSGDKN LIRVFQEGRD IHTETASWMF GVPREAVDPL MRRAAKTINF GVLYGMSAHR
481 LSQELAIPYE EAQAFIERYF QSFPKVRAWL KKTLEEGRRR GYVETLFGRR RYVPDLEARV
541 KSVREHAERM AFNMPVQGTA ADLMKLAMVK LFPRLEEMGA RMLLQVHDEL VLEAPKERAE
601 AVARLAKEVM EGVYPLAVPL EVEVGIGEDW LSAKG
```

```
R587Q = R390Q above [6MTaq# = DPO1_THEAQ# - 197]
E626K = E429K above [6MTaq# = DPO1_THEAQ# - 197]
I707L = I510L above [6MTaq# = DPO1_THEAQ# - 197]
E708K = E511K above [6MTaq# = DPO1_THEAQ# - 197]
A743H = A546H above [6MTaq# = DPO1_THEAQ# - 197]
E832G = E635G above [6MTaq# = DPO1_THEAQ# - 197]
```

A few amino acids were targeted because they fell into an evolutionary pattern of rapid change (heterotachy) or conserved-but-different. These patterns of evolution help the researcher to identify sites that are relevant enough to remain conserved in some subfamilies while at the same

time they tolerate some change, and this is reflected in the variability observed in another subfamily.

Analyzing amino acid sites became especially interesting when we examined them in the light of natural amino acid substitutions that have occurred at homologous sites during the natural history of natural polymerases. Two particular patterns of sequence change are often discussed, one that does not fit standard Markov models for amino acid replacement during functionally constrained protein evolution, the other that does. These models generally assume that future replacements are independent of past replacements. Replacement rates are characteristic of individual sites throughout evolutionary history, and amino acid probabilities at a site are characteristic of that site as well. These models are based on the assumption of stationarity in the functional roles of individual sites.

The first pattern is called conserved-but-different, "splits", or type-II functional divergence[1] among molecular evolutionists. It captures the Markovian assumptions and is identified by a site where an amino acid is conserved in (for example) two subfamilies, but the conserved amino acid differs between the two subfamilies (**Supplementary Fig. 14**). This difference may have an adaptive explanation, arguing that the difference reflects different function in the two subfamilies. Alternatively, it may simply reflect divergence at a site critical for core function (hence the relatively high level of conservation), but where (over long periods of natural history), changes at other sites allow the replacement without loss of fitness. In either case, the standard Markov model captures both the time and distribution of amino acid replacements that are observed.

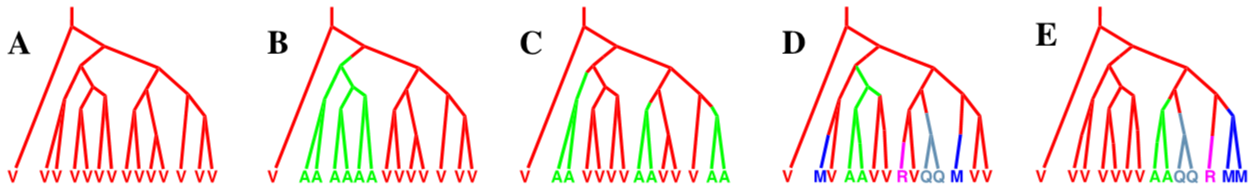

Supplementary Figure 14 | Different patterns of amino acid replacement shown schematically. **A**. A site where the amino acid is absolutely conserved; current protein engineering strategies leave this site alone, as any individual having a replacement in this site, throughout natural history, has evidently died. **B**. Constant-but-different. This pattern is captured by the standard Markov model for replacement; current strategies for protein engineering differ in its treatment, with "shuffling" strategies likely to encounter it. **C**. Homoplasy, where parallel changes have been made multiple times during natural history; current strategies for protein

engineering allow replacement, as long as the replacement amino acid is the same as seen in nature. **D**. Site with rapid divergence. Current protein engineering strategies can alter the amino acid at this site, but such alteration is unlikely to impact phenotype, as it seems to have not impacted phenotype during natural history to trigger much purifying selection. **E.** A site displaying heterotachy, with the rate of replacement much faster in the right subfamily than elsewhere.

The second pattern is called heterotachy, type-I functional divergence, or (by some) covarion-like behavior. This pattern involves differences in the rates of amino acid replacement at a site during different times/places of natural history (**Supplementary Fig. 14**). In some times/places, the site is strongly conserved; in others, it is less conserved[2-4]. Such sites are identified by observing that in one subfamily, amino acid replacement is frequent, while in another, amino acid replacement is infrequent (**Supplementary Fig. 14**). Adaptive explanations must be invoked to explain this non-Markovian behavior. A “selectionist” view would hold that in the subfamily where rapid replacement is seen, function is rapidly changing, allowing natural selection to “positively” select for amino acid replacements. A “neutralist” view would hold that the rapid change in one family reflects neutral drift of a site functionally unimportant in that subfamily, while functional constraints unique to the other subfamily required that purifying selection remove replacements that damaged a (possibly unknown) functionally important phenotype.

These two types of changes provide different instructions to the protein engineer. The first warns the engineer against making any replacement at that site. Although a replacement was evidently tolerated, it was tolerated only after long episodes of time, a time during which many (largely unknown) changes were required for tolerance. The second, however, invites a protein engineer to make changes. Either explanation for the heterotachy suggests that amino acid replacements at that site might alter an interesting phenotype, but also suggests that they can do so without destroying a core phenotype (like folding). The “neutralist” explanation points to the conserved branch as evidence for the first suggestion, and to the variable branch as evidence for the second. The “selectionist” explanation points to both branches as evidence for the first suggestion, and to the conserved branch as evidence for the second.

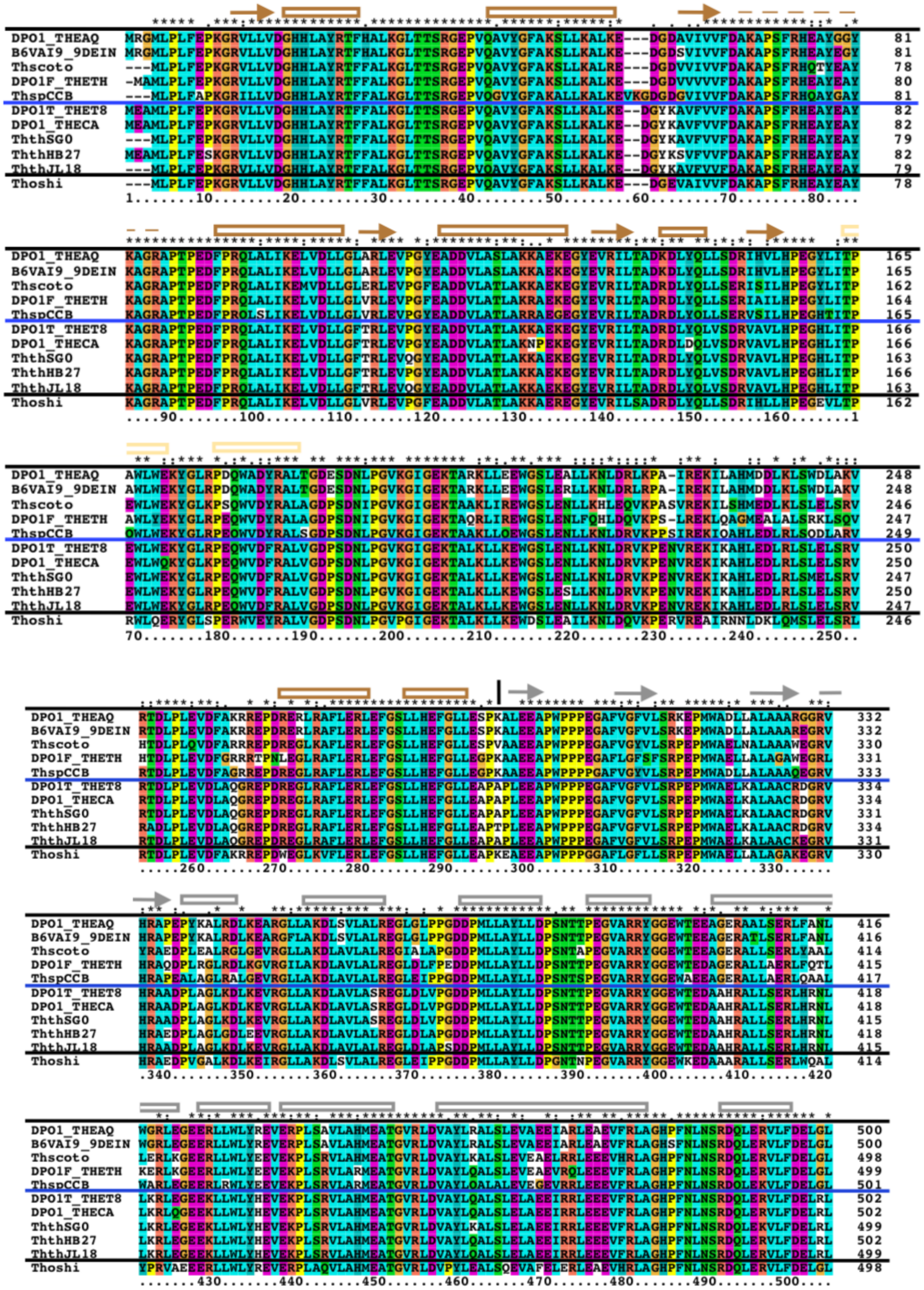

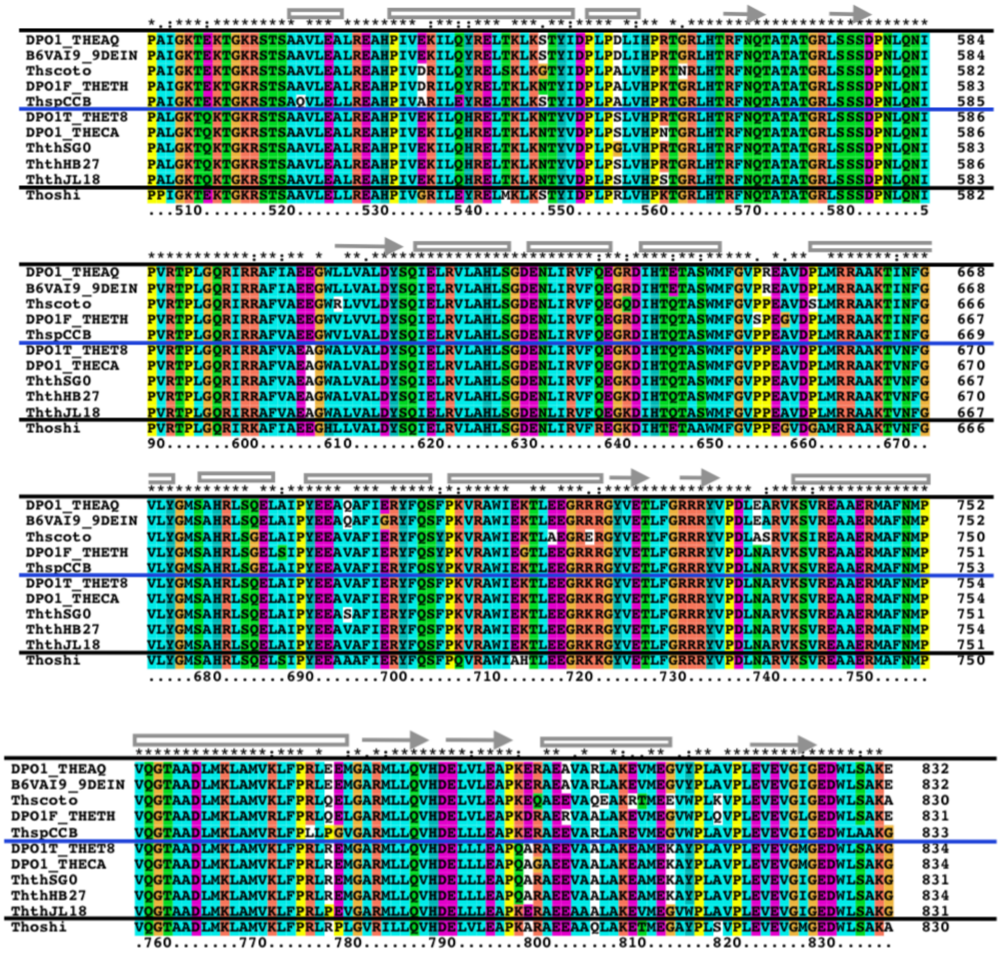

Supplementary Figure 15 | MSA of closest homologs of Taq polymerase. Top is DPO1_THEAQ is the Taq polymerase. DPO1T_THETH and DPO1T_THET8 both correspond to entries of Thermus thermophilus DNA polymerase.

Percent Identity Matrix - created by Clustal2.0.10

1: DPO1_THEAQ	100	99	86	86	88	87	86	87	87	88	84
2: B6VAI9_9DEIN	99	100	86	85	87	87	86	87	87	87	83
3: Thscoto	86	86	100	89	87	86	85	85	86	86	83
4: DPO1F_THETH	86	85	89	100	87	86	85	85	85	86	82
5: ThspCCB	88	87	87	87	100	86	85	86	86	87	82
6: DPO1T_THET8	87	87	86	86	86	100	99	99	99	98	84
7: DPO1_THECA	86	86	85	85	85	99	100	98	98	97	83
8: ThthSG0	87	87	85	85	86	99	98	100	98	98	83
9: ThthHB27	87	87	86	85	86	99	98	98	100	97	83
10: ThthJL18	88	87	86	86	87	98	97	98	97	100	83
11: Thoshi	84	83	83	82	82	84	83	83	83	83	100

Supplementary Figure 16 | Percent identity matrix of the sequences shown on Supplementary Fig. 15.

Moreover, heterotachy suggests that amino acid replacements at that site might alter an interesting phenotype, but also suggests that they can do so without destroying a core phenotype (like folding). The “neutralist” explanation points to the conserved branch as evidence for the first suggestion, and to the variable branch as evidence for the second. The “selectionist” explanation points to both branches as evidence for the first suggestion, and to the conserved branch as evidence for the second. Exploiting such sequence patterns in order to create polymerase variants that more efficiently search “sequence space”[5] seemed appropriate. Instead of randomly replacing amino acids in *Taq* DNA polymerase (which is theoretically expected to give, and empirically shown to give, an overwhelmingly large number of inactive proteins at the same time as many proteins having no detectable differences in phenotype), we considered heterotachy and CBD for “evolutionary guidance” as a way of constraining the search of sequence space. Here, we identified sites that displayed heterotachy and CBD, including those that have been previously examined to develop polymerases that incorporate **Z**:**P** pairs[6], as well as sites chosen using more conventional strategies.

The mutation R587Q came from semi-rational design and the mutation E832G came from the search for a secondary mutation to stabilize the first mutation. Structural information from evolved DNA polymerases[7] pointed to the site 587 in *Taq* polymerase as a potential site to avoid the negatively charged **Z** which would lead to the loss of the **Z:P** pair to C:G. In *Thermophilus* DNA Polymerase the mutations E628K; I709L; E710K; A745H are reported by Aye et al[8] to make *Taq* a hot start (E628K and I709L); while E710K provides resistant to inhibitors and A745H improves processivity.

Synthesis of 7-functionalized-dPTP derivatives

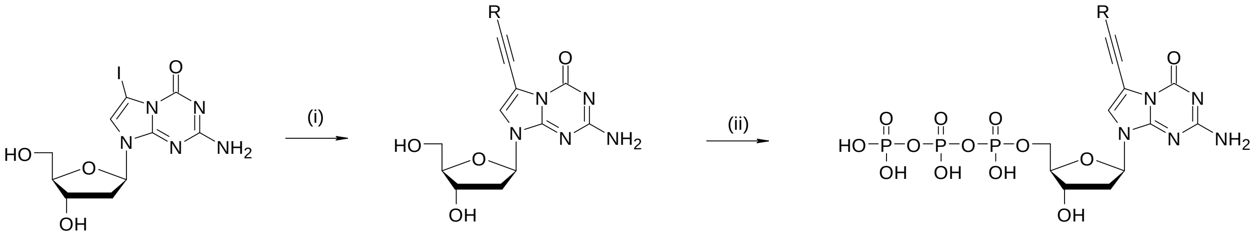

1 — **2a** (R=H), **2b** (R=CH_2CH_2Ph) — **3a** (R=H), **3b** (R=CH_2CH_2Ph)

Supplementary Figure 17 | Synthesis of triphosphates of 7- functionalized dPTP. Reagents and conditions: (i) (a) $Pd(PPh_3)_4$, CuI, Et_3N, trimethylsilylacetylene, room temperature, 12h (b) K_2CO_3, MeOH for **2a**; $Pd(PPh_3)_4$, CuI, Et_3N, 4-phenyl-1-butyne, room temperature, 12h for **2b**; (ii) (a) $POCl_3$, Triethyl phosphate, room temperature, 12 h, (b) tributylammonium pyrophosphate, 30 min.

2-Amino-8-(2-deoxy-β-D-erythro-pentofuranosyl)-7-ethynyl-imidazo[1,2-a]-1,3,5-triazin-4-(8H)-one (2a)

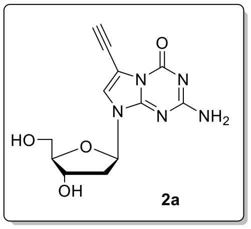

A solution consisting of 7-iodo-dP nucleoside[9] (**1**). (393 mg, 1 mmol), CuI (38 mg, 0.2 mmol), and $Pd(PPh_3)_4$ (116 mg, 0.1 mmol) in DMF (15 mL) was stirred at room temperature. Et_3N (0.35 mL, 2.5 mmol) and trimethylsilylacetylene (0.54 mL, 4 mmol) were added to the mixture. The resulting reaction mixture was stirred overnight at room temperature and then evaporated. The resulting residue was subjected to silica gel column chromatography (using CH_2Cl_2/MeOH as the eluent with a gradient of 20/1 to 6/1) to obtain the TMS-ethynyl derivative (210 mg, 0.58 mmol, 58%). The TMS-ethynyl derivative was then dissolved in MeOH (25 mL) and K_2CO_3 (44 mg, 0.32 mmol) was added to the solution. The reaction mixture was stirred for 1 h at room temperature, and then evaporated. The residue was again purified by silica gel column chromatography (using CH_2Cl_2/MeOH as the eluent with a gradient of 10/1 to 5/1) to give **2a** (157 mg, 0.54 mmol, 94%).

^{1}H NMR (DMSO-d_6, 300 MHz) δ 7.81 (s, 1H, -C=C*H*), 7.05 (brs, 2H, -N*H*$_2$), 6.12 (t, 1H, *J* = 6.6 Hz, H-1), 5.33 (d, 1H, *J* = 3.9 Hz, H-4), 5.02 (t, 1H, *J* = 5.4 Hz, H-3), 4.60 (s, 1H, -C≡C*H*), 4.31 (m, 1H, -O*H*), 3.79 (m, 1H, -O*H*), 3,49-3.58 (m, 2H, H-5a/5b), 2.33-2.42 (m, 1H, H-2a), 2.14-2.22 (m, 1H, H-2b). ^{13}C NMR (DMSO-d_6, 75 MHz) δ 168.4, 150.4, 150.1, 148.7, 121.2, 105.1, 88.2, 87.5, 83.4, 72.0, 70.6, 61.7. HRMS [ESI] calc. for $C_{12}H_{13}N_5NaO_4$ $[M+Na]^+$: 314.0860, found: 314.0865.

2-Amino-8-(2-deoxy-β-D-erythro-pentofuranosyl)-7-(4-phenyl-1-butynyl)-imidazo[1,2-a]-1,3,5-triazin-4-(8H)-one (2b)

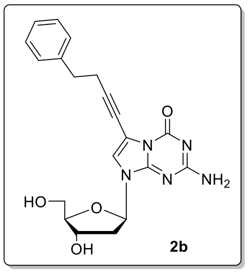

A stirred solution of 7-iodo-dP nucleoside (**1**) (1.12 g, 2.85 mmol), CuI (108 mg, 0.57 mmol), and $Pd(PPh_3)_4$ (330 mg, 0.29 mmol) in DMF (15 mL) was treated with Et_3N (1.14 mL, 8.18 mmol) and 4-phenyl-1-butyne (1.6 mL, 11.4 mmol) at room temperature. The reaction mixture was allowed to stir overnight at room temperature before being evaporated. The resulting residue was purified by silica gel column chromatography using

CH_2Cl_2/MeOH as the eluent with a gradient of 10/1 to 6/1, affording 2b (776 mg, 1.97 mmol, 69% yield).

^{1}H NMR (DMSO-d_6, 300 MHz) δ 7.52 (s, 1H, -C=C*H*), 7.15-7.31 (m, 5H, Ar-H), 6.95 (brs, 2H, -N*H*$_2$), 6.08 (t, 1H, *J* = 7.2 Hz, H-1), 4.25 (m, 1H, H-4), 3.74 (m, 1H, H-3), 3.45-3.50 (m, 2H, H-5a/b), 2.79-2.85 (m, 2H, -C*H*$_2$-), 2.66-2.71 (m, 2H, -C*H*$_2$-), 2.28-2.37 (m, 1H, H-2b), 2.10-2.17 (m, 1H, H-2b). ^{13}C NMR (DMSO-d_6, 75 MHz) δ 163.4, 150.3, 150.2, 149.0, 140.7, 129.6, 128.7, 126.7, 119.1, 113.7, 110.0, 106.2, 96.3, 88.1, 83.3, 70.7, 69.5, 61.7, 34.3, 21.6.HRMS [ESI] calc. for $C_{20}H_{21}N_5NaO_4$ $[M+Na]^+$: 418.1486, found: 418.1493.

2-Amino-8-(2-deoxy-β-D-erythro-pentofuranosyl)-7-ethynyl-imidazo[1,2-a]-1,3,5-triazin-4-(8H)-one 5’-Triphosphate (3a)

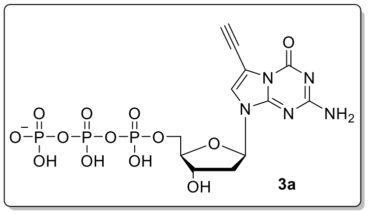

The synthesis of 5’-triphosphate involved the addition of $POCl_3$ (95 µl) to a stirred solution of **2a** (150 mg, 0.52 mmole) in triethylphosphate (4 mL) at 0°C. The reaction mixture was then stirred at 0°C for 3 hours, followed by overnight stirring at room temperature. A mixture of tributylammonium pyrophosphate (670 mg) and Bu_3N (0.7 mL) in CH_3CN (2 mL) was subsequently added, and the resulting mixture was allowed to react for 30 minutes. The reaction mixture was then diluted with water (30 mL) and extracted with CH_2Cl_2 (10 mL x 2). The aqueous layer was filtered through a 0.2 µm filter and subjected to purification by ion-exchange HPLC (Dionex BioLC DNAPac PA-100, 22 x 250 mm), using eluent A (water) and eluent B (1 M aq. NH_4HCO_3). The gradient was set to 0-50% B over 20 minutes, with a flow rate of 10 mL/min. The resulting product was further purified by reverse phase HPLC (HYPERSIL GOLD Prep C18 column, 5 µm, 20 x 250 mm), using eluent A (25 mM TEAA pH 7) and eluent B (50% CH_3CN in A). The gradient was set to 0-100% B over 20 minutes, with a flow rate of 10 mL/min. yielding the 5’-triphosphate (**3a**).

^{1}H NMR (D_2O, 300 MHz) δ 7.59 (s, 1H, -C=C*H*), 6.15 (t, 1H, *J* = 6.6 Hz, H-1), 4.55 (m, 1H, H-4), 3.95-4.06 (m, 3H, H5a/5b and H-3), 3.77 (s, 1H, -C≡C*H*), 2.39-2.47 (m, 1H, H-2a), 2.27-2.34 (m, 1H, H-2b). ^{31}P NMR (D_2O, 121. MHz) δ -10.02 (d, *J* = 19.5 Hz, 1P), -10.58 (d, *J* = 20.4 Hz, 1P), -22.40 (t, *J* = 19.9 Hz, 1P). HRMS [ESI] calc. for $C_{12}H_{15}N_5O_{13}P_3$ $[M]^-$: 529.9885, found: $[M]^-$ = 529.9885, $[M-HPO_3]^-$ = 450.0217.

2-Amino-8-(2-deoxy-β-D-erythro-pentofuranosyl)-7-(4-phenyl-1-butynyl)-imidazo[1,2-a]-1,3,5-triazin-4-(8H)-one 5’-Triphosphate (3b)

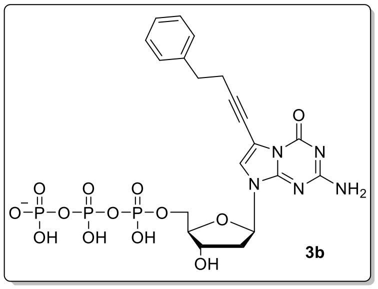

The synthesis of 5’-triphosphate (**3b**) involved the addition of $POCl_3$ (137 µl) at 0 °C to a stirred solution of **2b** (296 mg, 0.75 mmole) in triethylphosphate (4 mL). The reaction mixture was stirred at 0 °C for 3 hours, warmed to room temperature, and further stirred overnight. A mixture of tributylammonium pyrophosphate (1 g) and Bu_3N (1.05 mL) in CH_3CN (3 mL) was added to the reaction mixture and allowed to react for 30 minutes. Subsequently, the reaction mixture was diluted with water (40 mL) and extracted with CH_2Cl_2 (10 mL x 2). The aqueous layer was filtered through a 0.2 µm filter and subsequently purified by ion-exchange HPLC using a Dionex BioLC DNAPac PA-100 column (22 x 250 mm) with a gradient elution of eluent A (water) and eluent B (1 M aq. NH_4HCO_3) from 0 to 50% B over 20 minutes at a flow rate of 10 mL/min. Finally, reverse phase HPLC was conducted

using a HYPERSIL GOLD Prep C18 column (5 µm, 20 x 250 mm) with a gradient elution of eluent A (25 mM TEAA pH 7) and eluent B (50% CH_3CN in A) from 0 to 100% B over 20 minutes at a flow rate of 10 mL/min, yielding the 5'-triphosphate (**3b**).

^{1}H NMR (D_2O, 300 MHz) δ 7.10-7.32 (m, 6H, -C=C*H* and Ar-H), 6.11 (m, 1H, H-1), 4.55 (m, 1H, H-4), 4.03 (m, 3H, H5a/5b and H-3), 2.73 (m, 2H, -CH_2-), 2.57 (m, 2H, -CH_2-), 2.38 (m, 1H, H-2a), 2.26 (m, 1H, H-2b). ^{31}P NMR (D_2O, 121 MHz) δ -9.93 (d, *J* = 19.5 Hz,1P), -10.50 (d, *J* = 19.6 Hz, 1P), -22.28 (t, *J* = 19.8 Hz, 1P). HRMS [ESI] calc. for $C_{20}H_{23}N_5O_{13}P_3$ $[M]^-$: 634.0511, found: $[M]^-$ = 634.0500, $[M-HPO_3]^-$ = 554.0856.

HPLC and mass spectrometry of ZZ sequence.

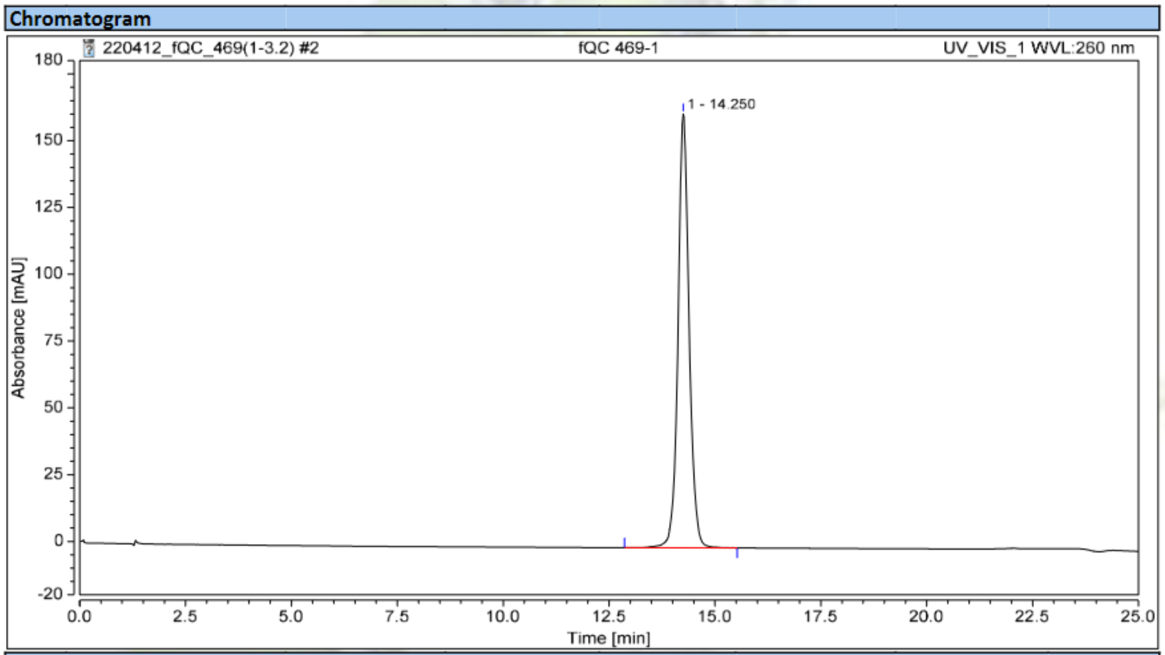

Integration Results

No.	Peak Name	Retention Time min	Area mAU*min	Height mAU	Relative Area %	Relative Height %	Amount n.a.
1		14.250	49.179	162.398	100.00	100.00	n.a.
**Total:**			**49.179**	**162.398**	**100.00**	**100.00**	

Supplementary Figure 18 | Analysis of ZZ sequence by HPLC.

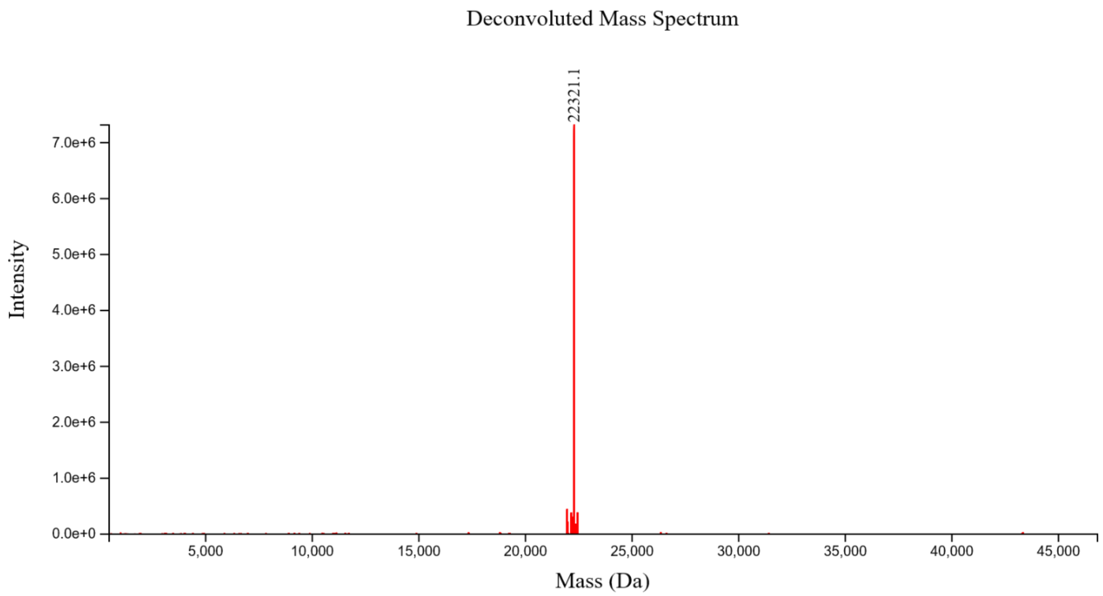

Supplementary Figure 19 | Analysis of ZZ sequence by mass spectrometry [ESI]. Calc. MW: 22320.5. Obs. Mass: 22321.1.

HPLC and mass spectrometry of ZP-1 sequence.

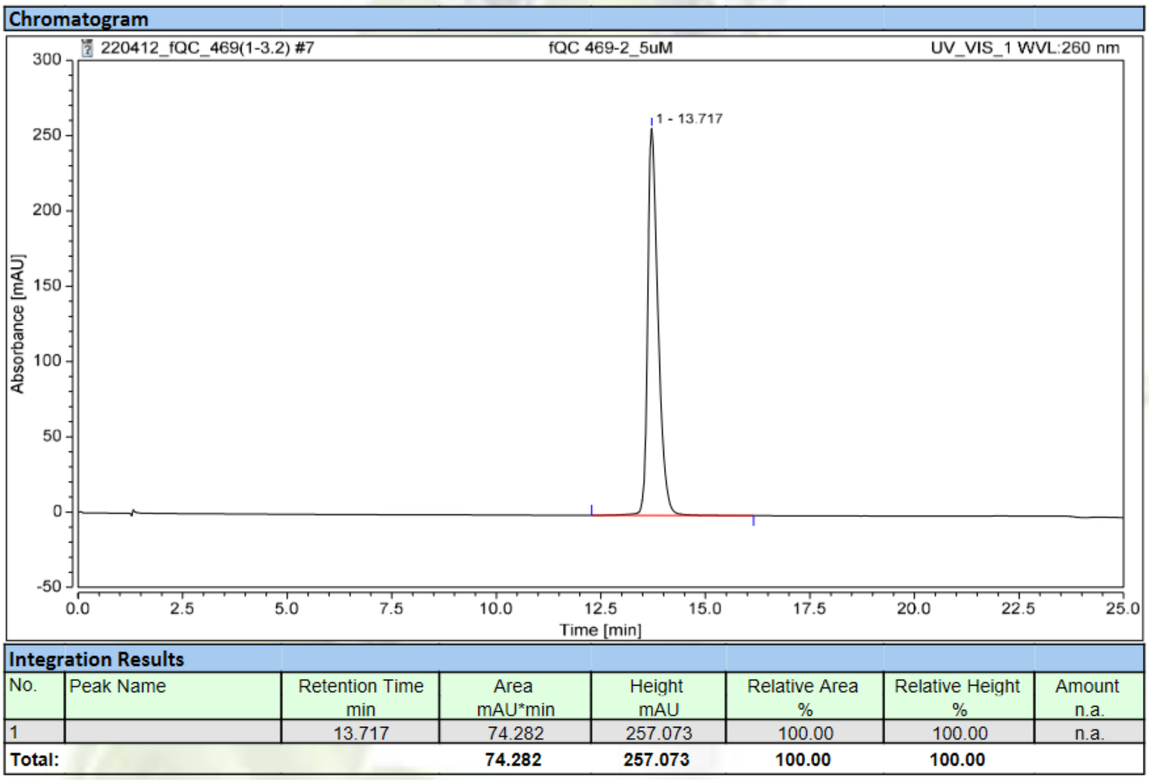

Integration Results

No.	Peak Name	Retention Time min	Area mAU*min	Height mAU	Relative Area %	Relative Height %	Amount n.a.
1		13.717	74.282	257.073	100.00	100.00	n.a.
**Total:**			**74.282**	**257.073**	**100.00**	**100.00**	

Supplementary Figure 20 | Analysis of ZP-1 sequence by HPLC.

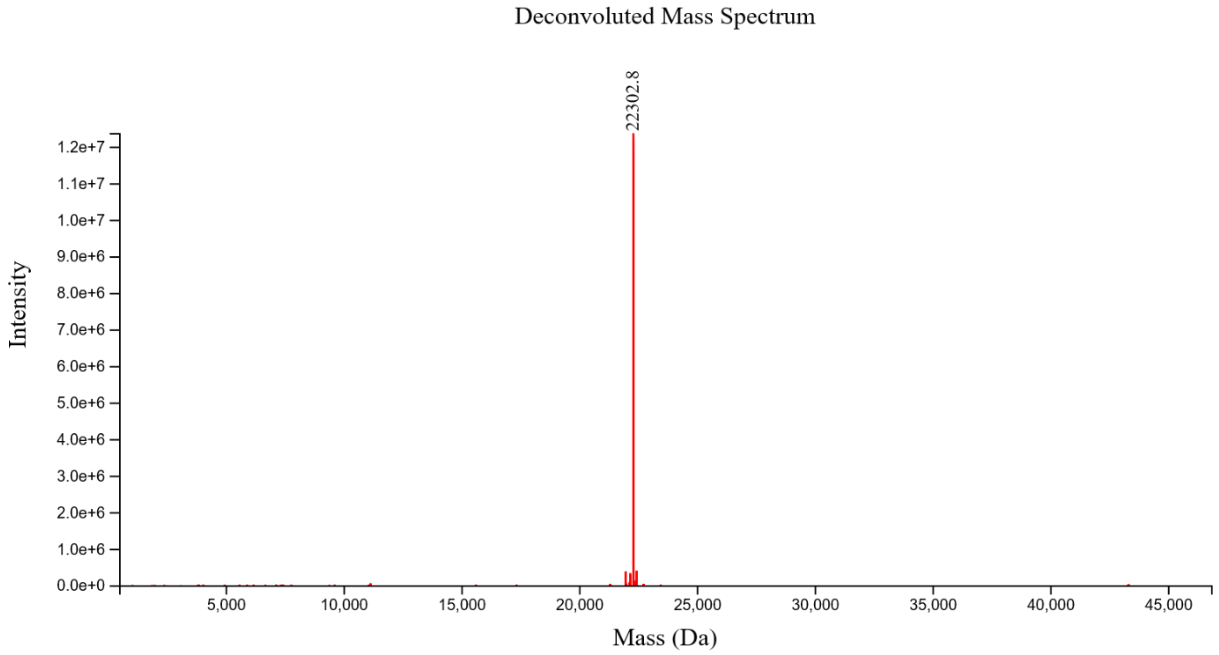

Supplementary Figure 21 | Analysis of ZP-1 sequence by mass spectrometry [ESI]. Calc. MW: 22302.5. Obs. Mass: 22302.8.

HPLC and mass spectrometry of ZP-2 sequence.

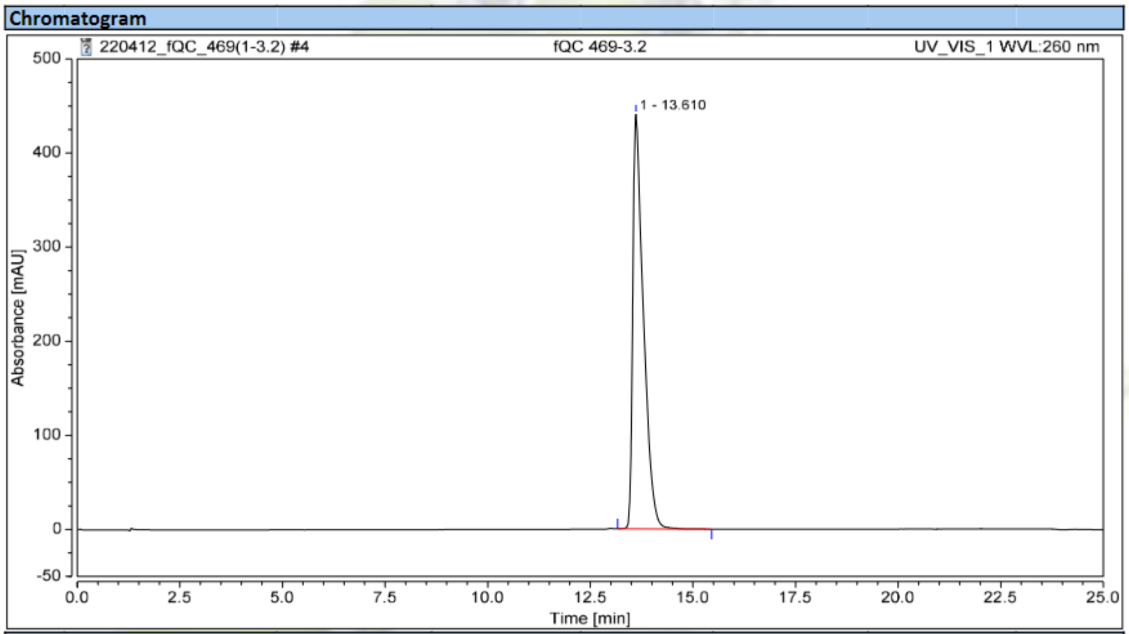

Integration Results

No.	Peak Name	Retention Time min	Area mAU*min	Height mAU	Relative Area %	Relative Height %	Amount n.a.
1		13.610	132.306	440.163	100.00	100.00	n.a.
**Total:**			**132.306**	**440.163**	**100.00**	**100.00**	

Supplementary Figure 22 | Analysis of ZP-2 sequence by HPLC.

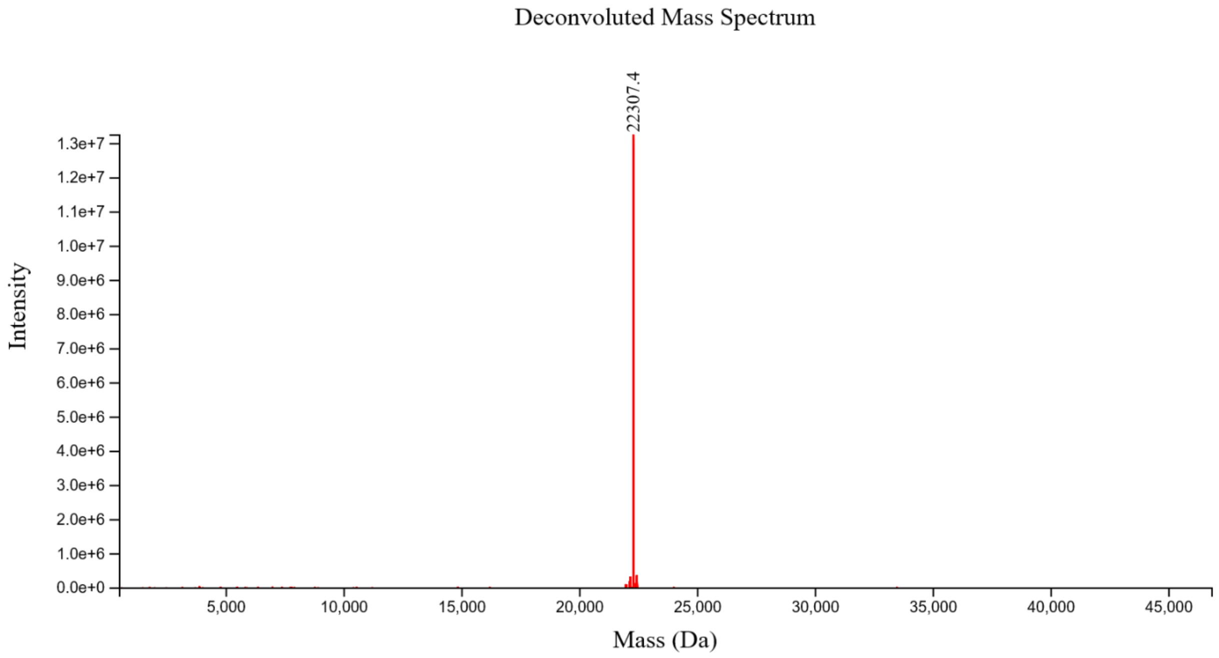

Supplementary Figure 23 | Analysis of ZP-2 sequence by mass spectrometry [ESI]. Calc. MW: 22307.5. Obs. Mass: 22307.4.

Analysis of Z-Ran sequence by HPLC.

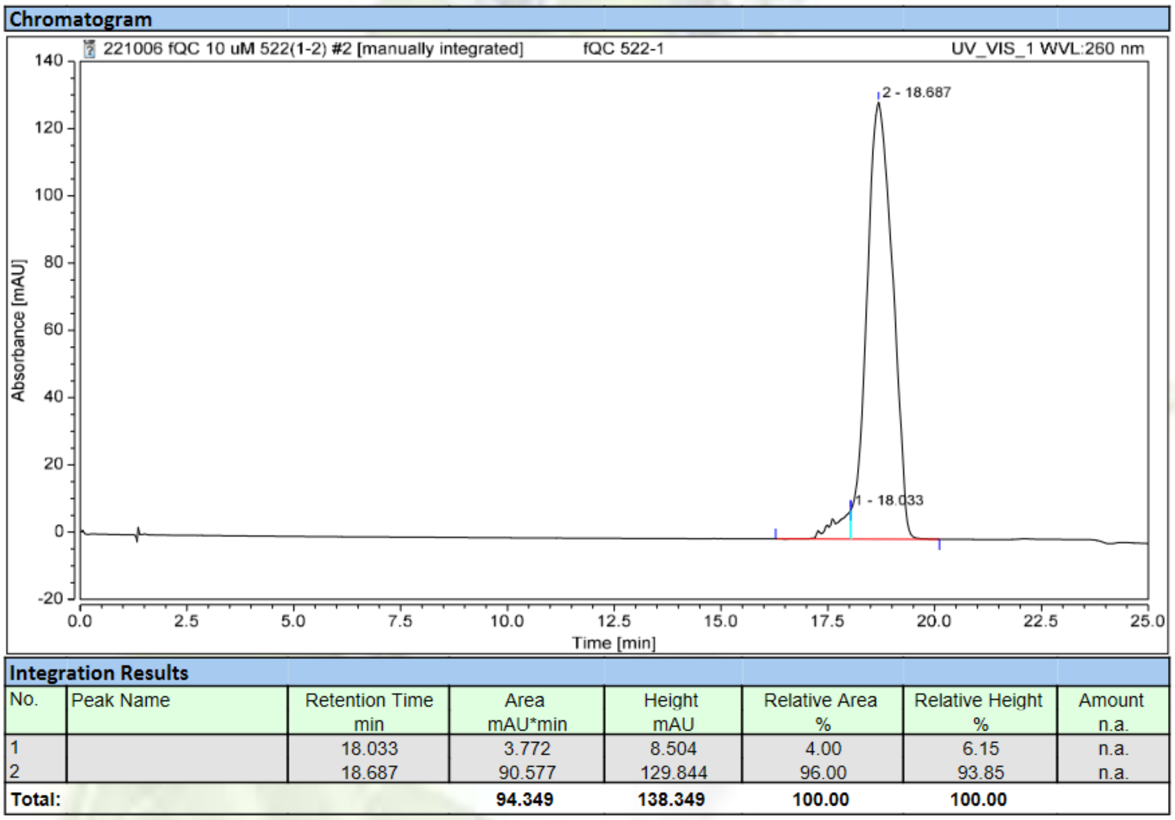

Integration Results

No.	Peak Name	Retention Time min	Area mAU*min	Height mAU	Relative Area %	Relative Height %	Amount n.a.
1		18.033	3.772	8.504	4.00	6.15	n.a.
2		18.687	90.577	129.844	96.00	93.85	n.a.
**Total:**			**94.349**	**138.349**	**100.00**	**100.00**	

Supplementary Figure 24 | Analysis of Z-Ran sequence by HPLC.

Analysis of P-Ran sequence by HPLC.

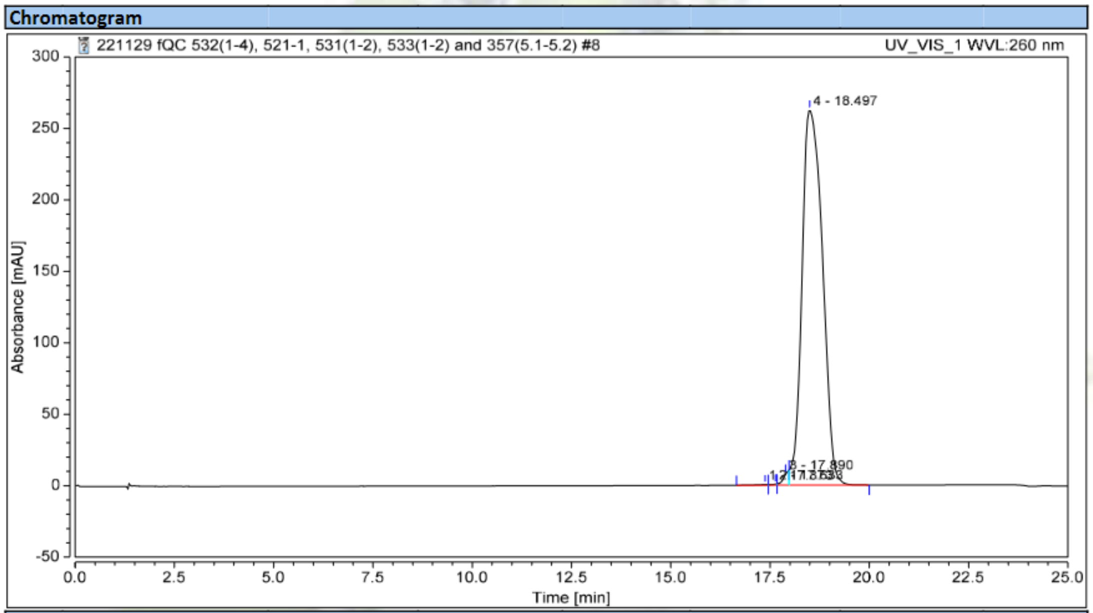

Integration Results

No.	Peak Name	Retention Time min	Area mAU*min	Height mAU	Relative Area %	Relative Height %	Amount n.a.
1		17.373	0.161	0.492	0.10	0.18	n.a.
2		17.633	0.122	0.731	0.08	0.27	n.a.
3		17.890	1.457	7.616	0.92	2.81	n.a.
4		18.497	156.699	262.537	98.90	96.74	n.a.
**Total:**			**158.439**	**271.376**	**100.00**	**100.00**	

Supplementary Figure 25 | Analysis of P-Ran sequence by HPLC.

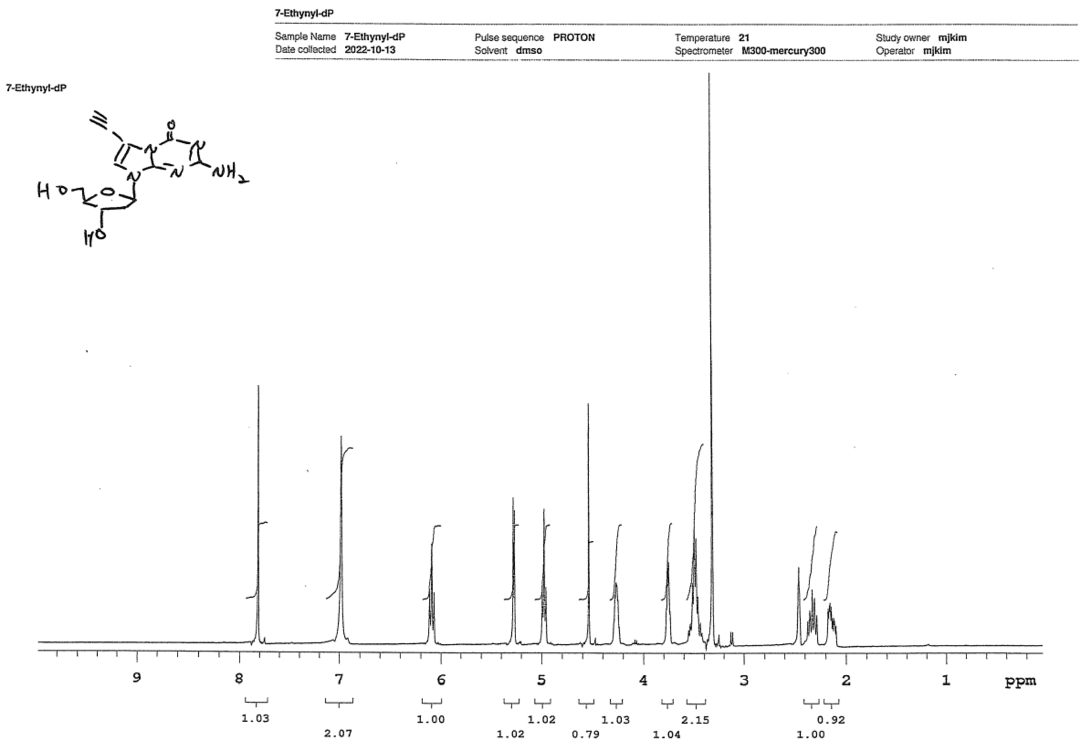

Supplementary Figure 26 | ^{1}H NMR spectrum of **2a** in DMSO, 300 MHz.

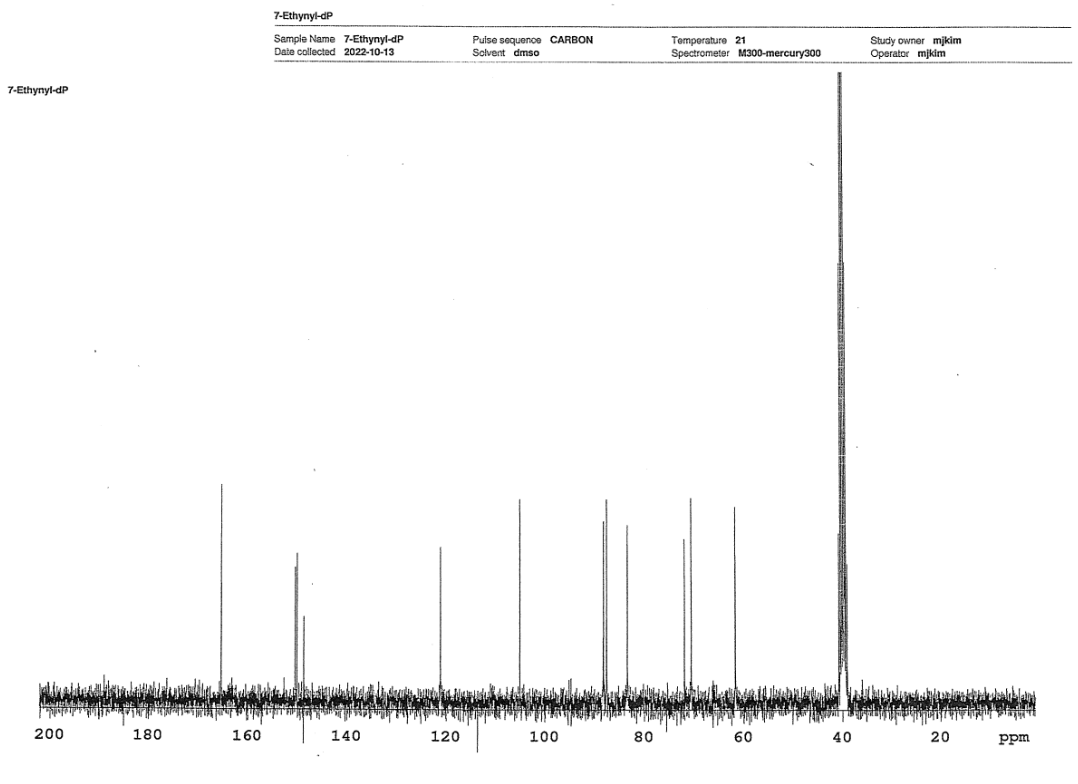

Supplementary Figure 27 | ^{13}C NMR spectrum of **2a** in DMSO, 75 MHz.

Mass Spectrometry Analysis Report

University of Florida - Mass Spectrometry Research and Education Center
Department of Chemistry
P.O. Box 117200, University of Florida, Gainesville, FL 32611-7200

UF Sequence # 33338-1
Name: Bang Wang
Advisor/Affiliation Name: Foundation for Applied Molecular Evolution (FFAME)
Sample Name: A1
Date Submitted: 03/06/2023
Date Analyzed: 3/14/2023
Analyzed by – Laura Bailey

Experimental setup -
Ionization Source: Electro spray ionization (ESI) (analyzed in positive mode)
small mass optimized (m/z 50 – 500)
Mass Spectrometry: Bruker Daltonics, Impact II QTOF
Drying gas (N_2) – 4 L/min, Gas temperature - 200˚C, Capillary – 4500 V, Nebulizer – 0.3 Bar
Injection System: Thermo UltiMate 3000 series system
Injection volume – 3 µL
Mobile Phase(s): 50% B (flow inject)
(A) 60/40 acetonitrile/water with 10 mM ammonium formate and 0.1% formic acid
(B) 90/8/2 isopropanol/acetonitrile/water with 10 mM ammonium formate and 0.1% formic acid
Flow Rate: NC pump- 10 µL/min

Sample Preparation:
Sample (trace) was dissolved in 1 mL methanol in a 1.5 mL glass autosample vial. Dilute sample was then analyzed by flow injection on the QTOF-MS. A plug of sodium formate was injected prior to the sample to allow re-calibration prior to analysis. Samples were stored at 4C when not in use.
Molecular Formula (M): C12 H13 N5 O4 Molecular weight: 291.0968 Da

Summary of Analysis

Masses related to the sample were observed: $[M+H]^+$ and $[M+Na]^+$

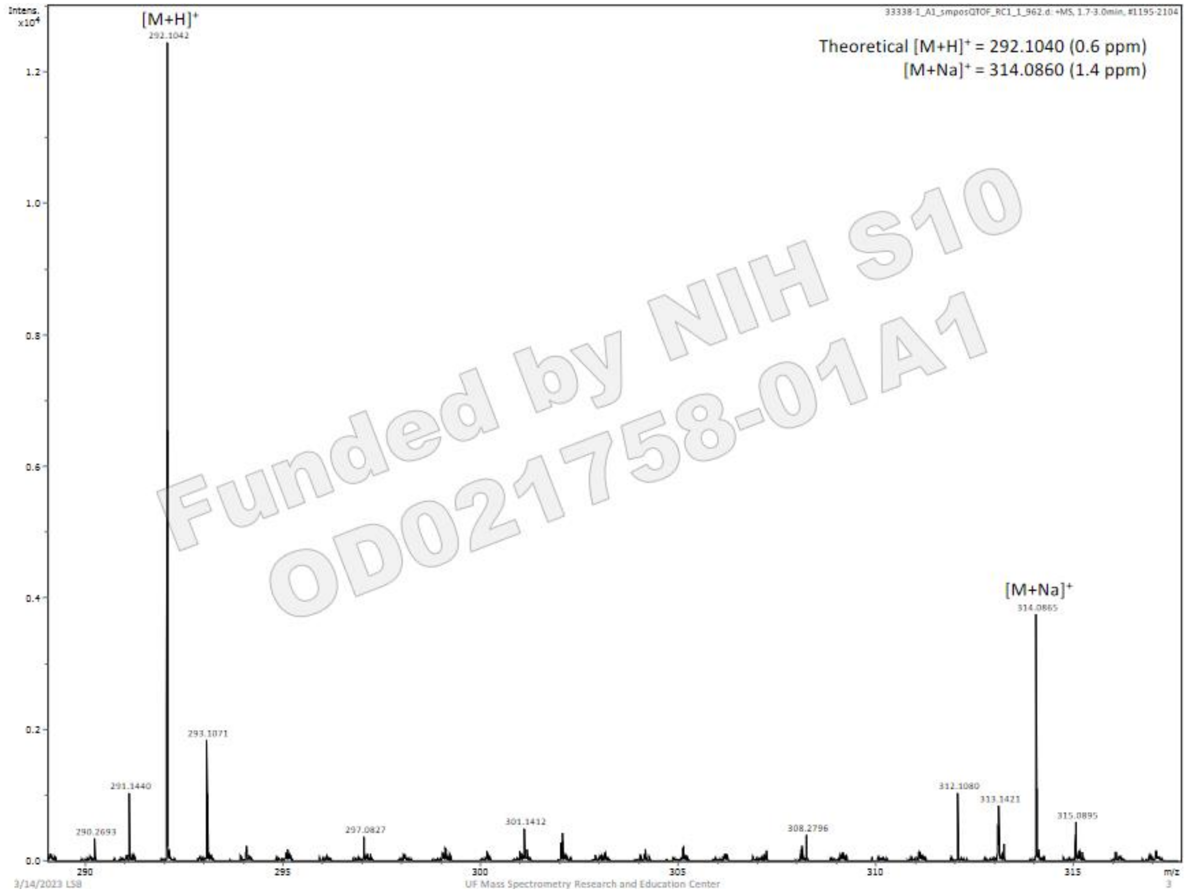

Funded by NIH S10 OD021758-01A1

Supplementary Figure 28 | HRMS for 2a,

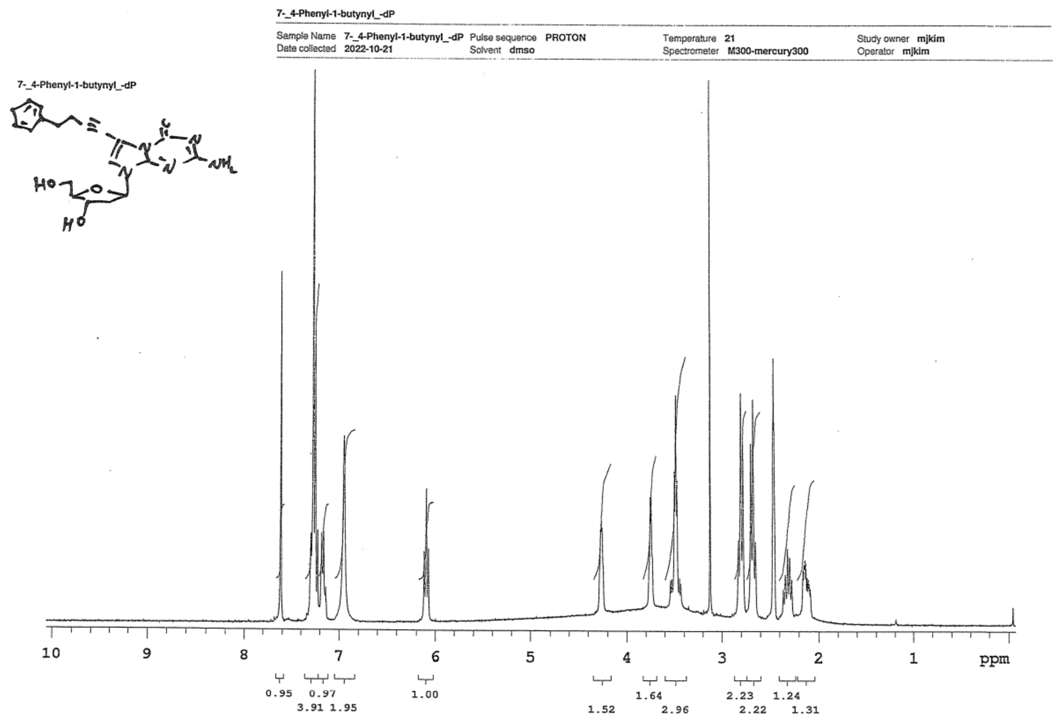

Supplementary Figure 29 | ^{1}H NMR spectrum of **2b** in DMSO, 300 MHz.

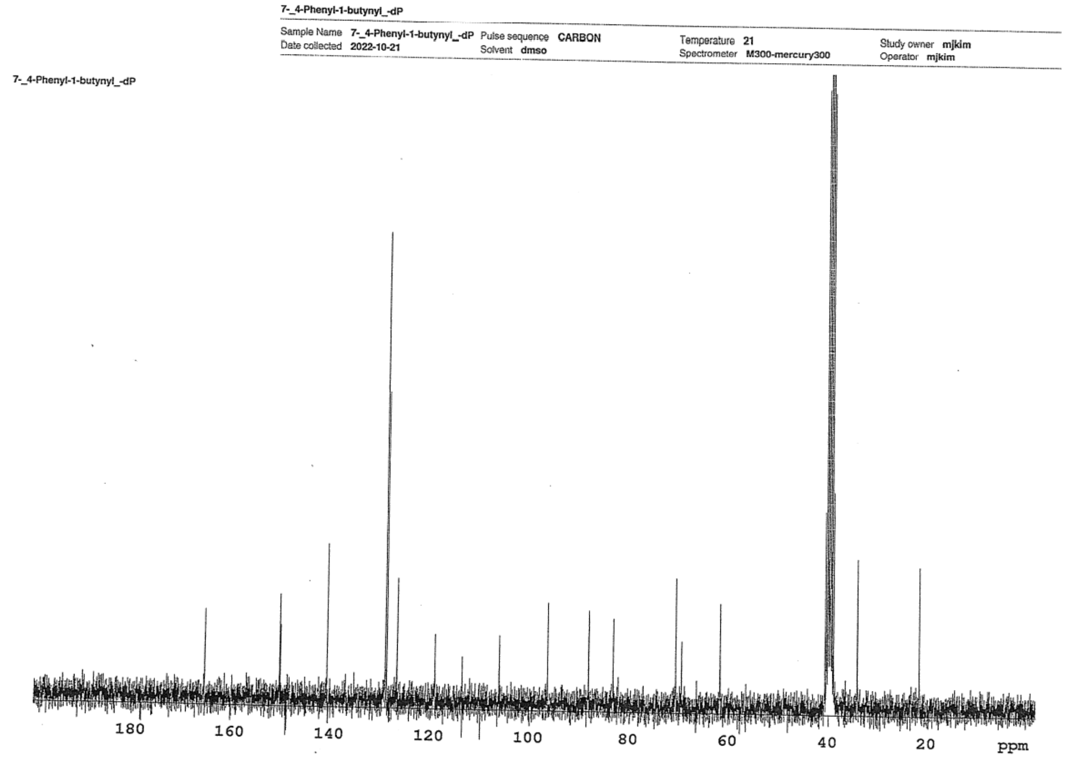

Supplementary Figure 30 | ^{13}C NMR spectrum of **2b** in DMSO, 75 MHz.

Mass Spectrometry Analysis Report

University of Florida - Mass Spectrometry Research and Education Center
Department of Chemistry
P.O. Box 117200, University of Florida, Gainesville, FL 32611-7200

UF Sequence # **33338-2**
Name: **Bang Wang**
Advisor/Affiliation Name: Foundation for Applied Molecular Evolution (FFAME)
Sample Name: **A2**
Date Submitted: 03/06/2023
Date Analyzed: 3/14/2023
Analyzed by – **Laura Bailey**

Experimental setup -
Ionization Source: Electro spray ionization (ESI) (analyzed in positive mode)
mid mass optimized (m/z 250 – 2500)
Mass Spectrometry: Bruker Daltonics, Impact II QTOF
Drying gas (N_2) – 4 L/min, Gas temperature - 200˚C, Capillary – 4500 V, Nebulizer – 0.3 Bar
Injection System: Thermo UltiMate 3000 series system
Injection volume – 1 µL
Mobile Phase(s): **50% B (flow inject)**
(A) 60/40 acetonitrile/water with 10 mM ammonium formate and 0.1% formic acid
(B) 90/8/2 isopropanol/acetonitrile/water with 10 mM ammonium formate and 0.1% formic acid
Flow Rate: NC pump- 10 µL/min

Sample Preparation:
Sample (trace) was dissolved in 1 mL methanol in a 1.5 mL glass autosample vial. Dilute sample was then analyzed by flow injection on the QTOF-MS. A plug of sodium formate was injected prior to the sample to allow re-calibration prior to analysis. Samples were stored at 4C when not in use.
Molecular Formula (M): C20 H21 N5 O4 Molecular weight: 395.1594 Da

Summary of Analysis

Masses related to the sample were observed: [M+H]$^+$ and [M+Na]$^+$

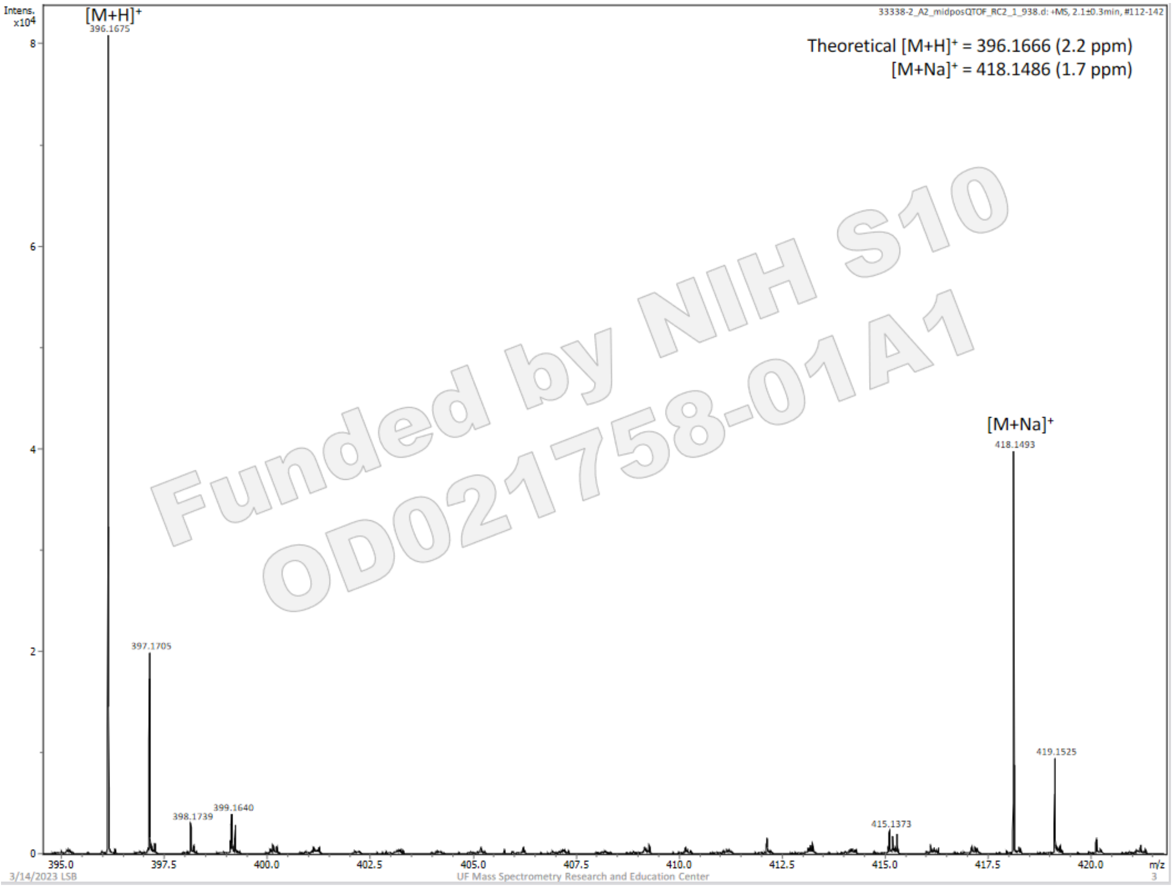

Supplementary Figure 31 | HRMS for 2b,

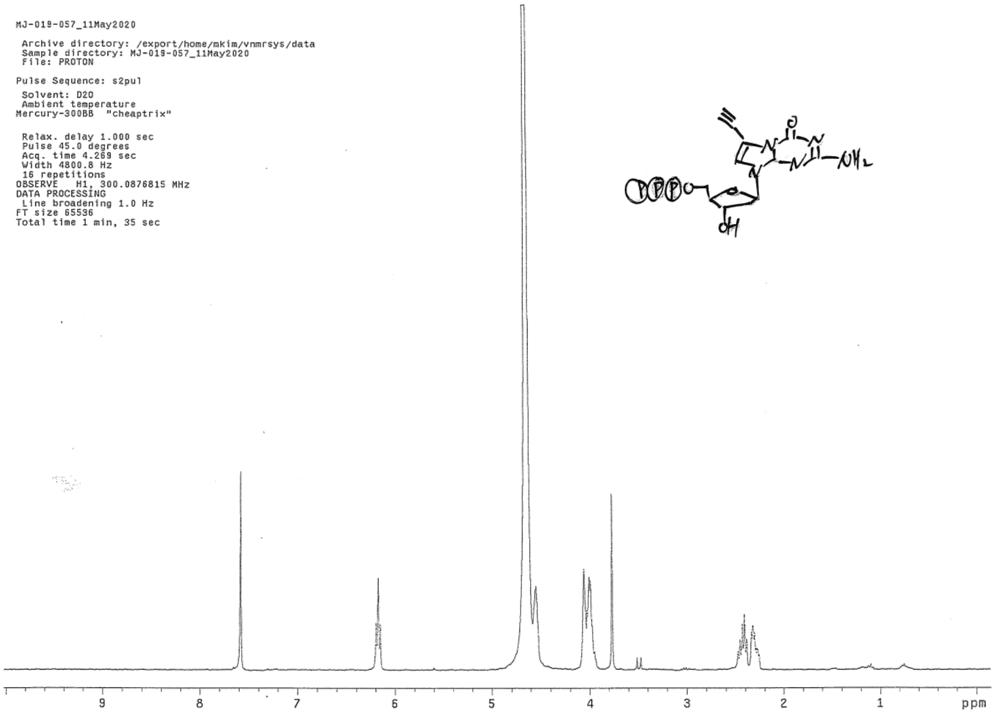

Supplementary Figure 32 | ^{1}H NMR spectrum of **3a** in D_2O, 300 MHz.

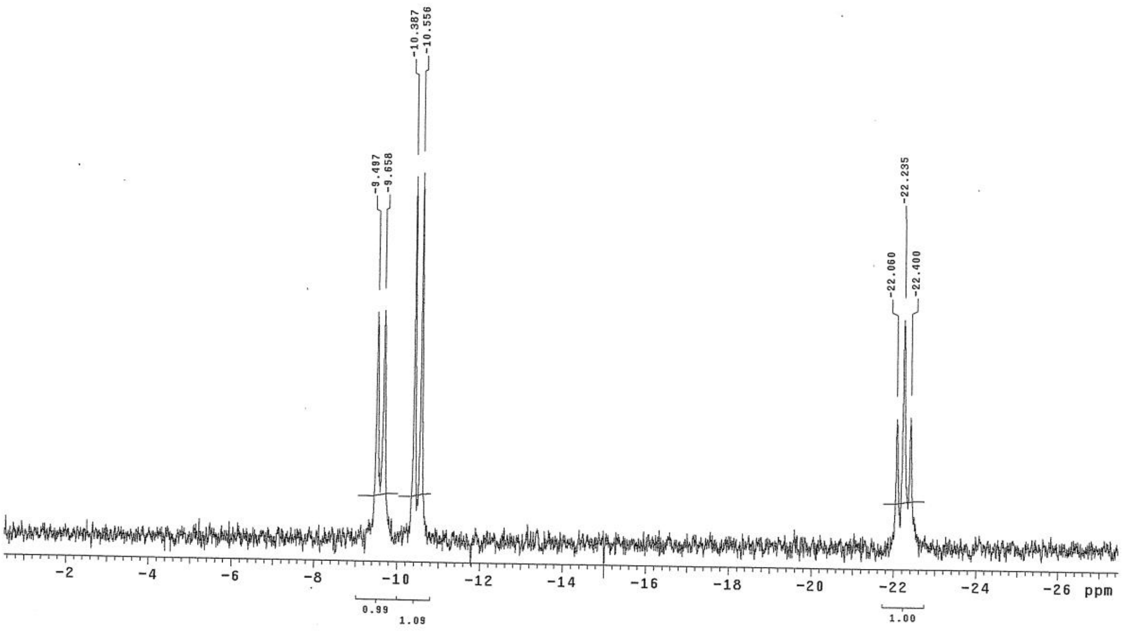

Supplementary Figure 33 | ^{31}P NMR spectrum of **3a** in D_2O, 121 MHz.

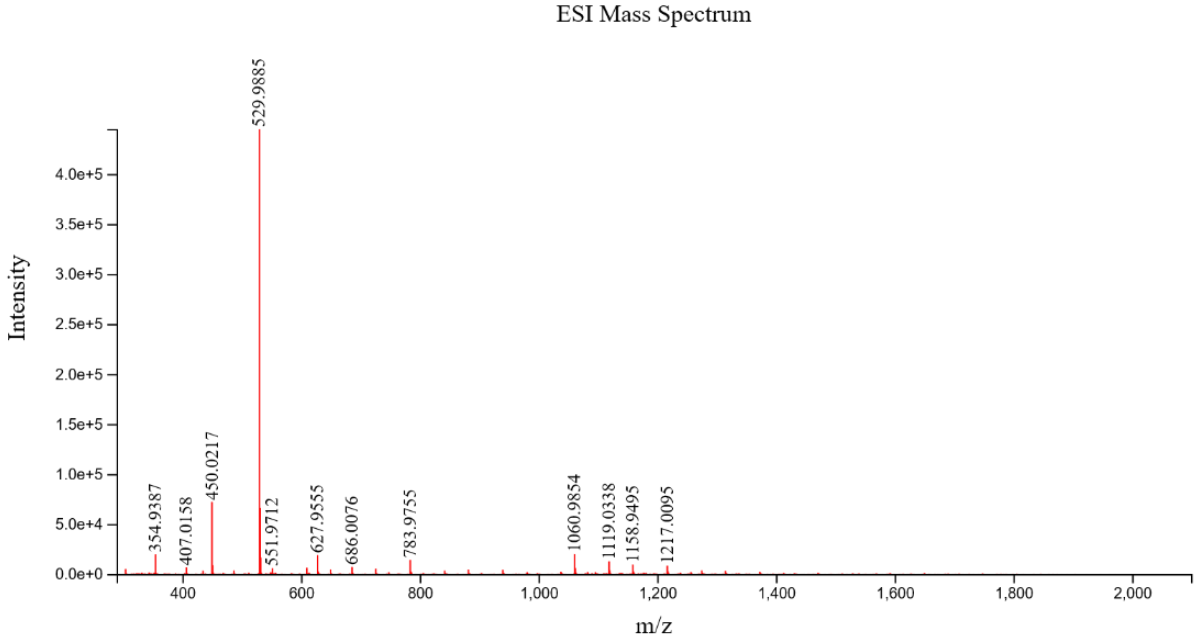

Supplementary Figure 34 | HRMS for 3a. Calc. $[M]^-$ = 529.9885; Obs. $[M]^-$ = 529.9885, $[M-HPO_3]^-$ = 450.0217.

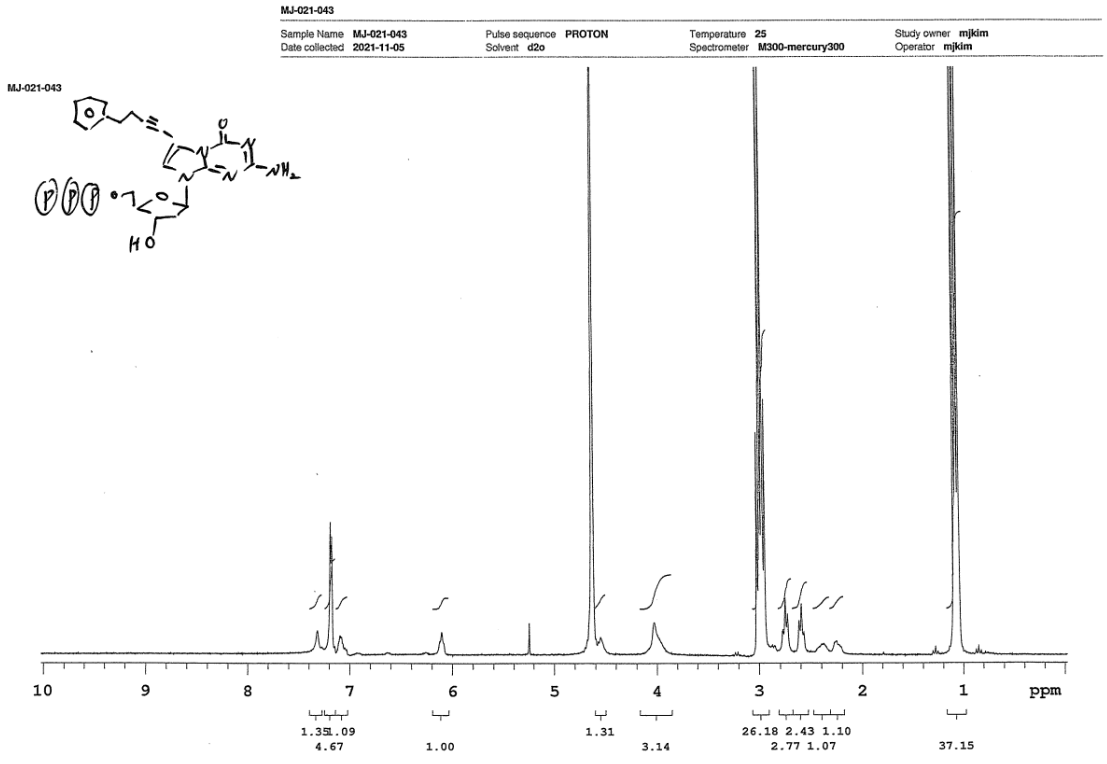

Supplementary Figure 35 | ^{1}H NMR spectrum of **3b** in D_2O, 300 MHz.

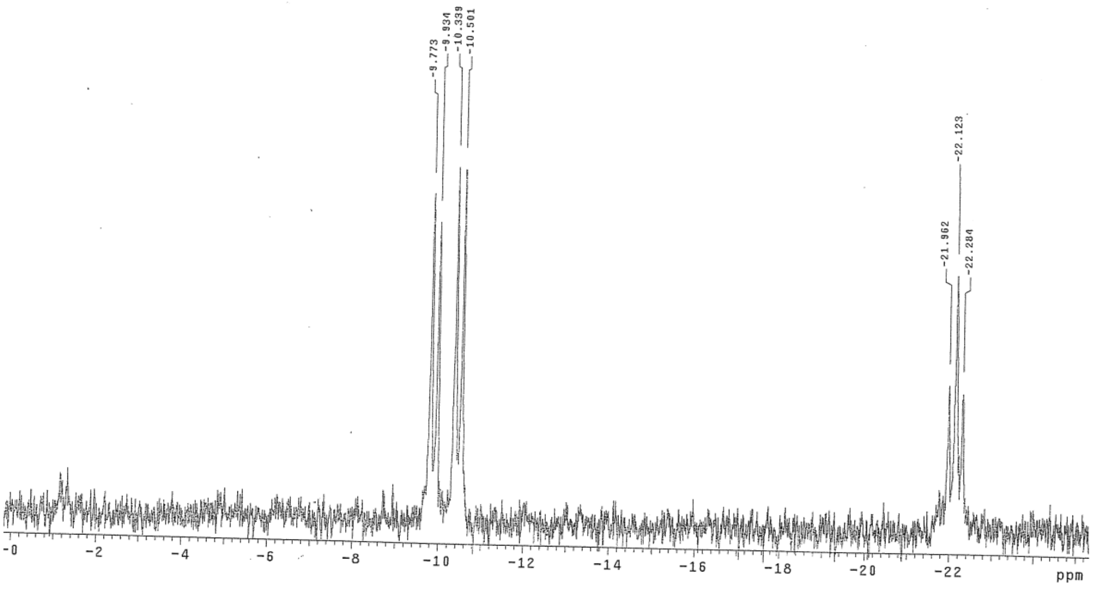

Supplementary Figure 36 | ^{31}P NMR spectrum of **3b** in D_2O, 121 MHz.

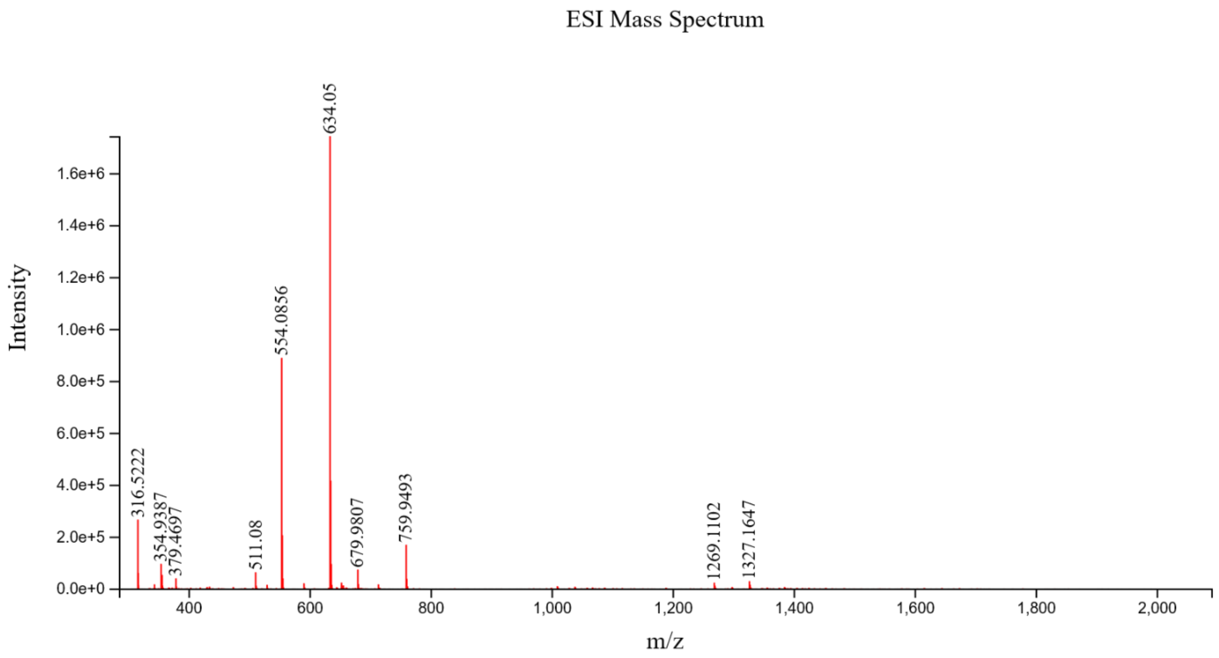

Supplementary Figure 37 | HRMS for 3b. Calc. $[M]^-$ = 634.0511; Obs. $[M]^-$ = 634.0500, $[M\text{-}HPO_3]^-$ = 554.0856.

Supplementary Note 1: The C# code used to detail the percentage of bases from a SAMTools pileup file.

```
/// <summary>
/// Input is a file made using the "mpileup" option in samtools.  It has the
following columns:
/// [0] Sequence Name, [1] Position, [2] Original base (will be N if no
"expected" sequence database used),
/// [3] Number of reads, [4] bases seen, [5] quality scores
///
/// File is processed to find runs of sequences above a certain size and
depth of coverage.
/// producing an output file with the following information:
/// [0] Start position, [1] End position, [2] average coverage, [3] coverage
min, [4] coverage max,
/// [5] average accuracy, [6] sequence
/// </summary>
/// <param name="fileNamewPath">Input file in mpileup format with full
path</param>
/// <param name="outputFilewPath">Output file to be produced with full
path</param>
/// <param name="positionStart">Starting position of sequence(s) to
process</param>
/// <param name="positionEnd">Ending position of sequence(s) to
process</param>
/// <param name="numMismatchesToDisplay">Total number of mismatching bases to
display in output</param>
public void GetSequenceInformationFromSAMPileupFile(string fileNamewPath,
string outputFilewPath,
    int positionStart = 0, int positionEnd = int.MaxValue, int
numMismatchesToDisplay = 3) {
    StreamReader myRdr = new StreamReader(fileNamewPath);
    string thisLine;
    string[] splitArr;
    int i;
    string[] savedLines = null;
    int savedPos = 0;
    int minSeqSize = 2;
    int minCoverage = 1;
    int splitCheckThreshold = 10;
    int maxCoverage = -1;
    int thisCoverage;
    int thisPosition;
    int prevPosition = -1;
    string prevSequence = "";
    string[,] positionArr = null;

    //Read file and store data to tracking arrays:
    while (myRdr.Peek() > -1) {
        thisLine = myRdr.ReadLine();
        splitArr = thisLine.Split('\t');
        if (splitArr.Length == 6) {
            thisCoverage = Convert.ToInt32(splitArr[3]);
```

```
            if (thisCoverage >= minCoverage && (maxCoverage <
splitCheckThreshold * 3 ||
                thisCoverage > splitCheckThreshold)) {
                //Make sure we are above minimum coverage and we are not in a
short
                //area between two close reads
                //  (i.e. an area that goes from 2000 read coverage to 4
reads and then back to 1000 reads.)
                thisPosition = Convert.ToInt32(splitArr[1]);
                if (thisPosition == prevPosition + 1 && splitArr[0] ==
prevSequence) {
                    if (savedPos >= savedLines.Length) {
                        Array.Resize(ref savedLines, savedLines.Length +
5000);
                    }
                    savedLines[savedPos] = thisLine;
                    prevPosition = thisPosition;
                    prevSequence = splitArr[0];
                    if (thisCoverage > maxCoverage) maxCoverage =
thisCoverage;
                    savedPos += 1;
                } else {
                    AddFoundSequenceToIndividualPositionArray(ref
positionArr, savedLines, savedPos,
                        minSeqSize, numMismatchesToDisplay, positionStart,
positionEnd);
                    savedLines = new string[4000];
                    savedLines[0] = thisLine;
                    savedPos = 1;
                    maxCoverage = -1;

                    prevPosition = thisPosition;
                    prevSequence = splitArr[0];
                }
            }
        }
    }
    //Store final records to arrays:
    AddFoundSequenceToIndividualPositionArray(ref positionArr, savedLines,
savedPos, minSeqSize,
        numMismatchesToDisplay, positionStart, positionEnd);
    myRdr.Close();

    //Create output file:
    StreamWriter outWtr = new StreamWriter(outputFilewPath +
".positions.tsv");
    string[] headerArr = new string[] { "Ref Name", "Base", "Position",
"Coverage", "Avg. Quality" };
    for (i = 0; i <= numMismatchesToDisplay; i++) {
        Array.Resize(ref headerArr, headerArr.Length + 2);
        headerArr[headerArr.Length - 2] = "Base " + (i + 1).ToString();
        headerArr[headerArr.Length - 1] = "Base " + (i + 1).ToString() + "
%";
```

```
    }
    outWtr.WriteLine(String.Join("\t", headerArr));

    if (positionArr != null) {
        for (i = 0; i < positionArr.GetLength(1); i++) {
            for (int j = 0; j < positionArr.GetLength(0) - 1; j++) {
                if (j != 5) {
                    //Skip the large pileup column to make a readable file:
                    outWtr.Write(positionArr[j, i] + "\t");
                }
            }
            outWtr.Write(positionArr[positionArr.GetLength(0) - 1, i] +
"\r\n");
        }
    }

    outWtr.Close();
}
/// <summary>
/// Creates individual record for each position in existing array with the
following positions:
/// [0] refSeq, [1] Base, [2] Position, [3] Coverage, [4] Avg. Quality, [5]
Most Common, [6] Match %,
/// [7] Largest mismatch base, [8] largest mismatch percent, [9] normalized
pileup
/// </summary>
/// <param name="posArr">Existing position array to append values</param>
/// <param name="savedLines">Array of lines saved for sequence from a pileup
file</param>
/// <param name="savedPos">Position of last line in the savedLines
array</param>
/// <param name="minSeqSize">The minimum sequences size to store</param>
/// <param name="numMismatchesToDisplay">The number of mismatching bases to
display in the output</param>
/// <param name="positionStart">The starting position of each sequence to
display in the output</param>
/// <param name="positionEnd">The ending position of each sequence to display
in the output</param>
public void AddFoundSequenceToIndividualPositionArray(ref string[,] posArr,
string[] savedLines,
    int savedPos, int minSeqSize, int numMismatchesToDisplay, int
positionStart = 0,
    int positionEnd = int.MaxValue) {
    int thisPos;
    string[] splitArr;
    int i;
    int j;
    int accurateSum;
    string thisBase;
    int seqPosition;
    bool ignoreGaps = true;
```

```
    if (savedPos >= minSeqSize) {
        if (posArr == null) {
            thisPos = 0;
            posArr = new string[8 + numMismatchesToDisplay * 2, savedPos];
        } else {
            thisPos = posArr.GetLength(1);
        }

        for (i = 0; i < savedPos; i++) {
            splitArr = savedLines[i].Split('\t');
            splitArr[2] = splitArr[2].ToUpper();
            seqPosition = Convert.ToInt32(splitArr[1]);
            if (seqPosition >= positionStart && seqPosition <= positionEnd) {
                thisBase = GetNthMostCommonBaseFromPileupString(splitArr[4],
1, splitArr[2], ignoreGaps);

                posArr[0, thisPos] = splitArr[0];
                posArr[1, thisPos] = splitArr[2];
                posArr[2, thisPos] = splitArr[1];
                posArr[3, thisPos] = splitArr[3];
                posArr[4, thisPos] =
                    Math.Round(GetAverageQualityScoreFromString(splitArr[5],
true), 2).ToString();
                posArr[5, thisPos] = GetNormalizedPileupString(splitArr[4],
true, ignoreGaps, splitArr[2]);
                for (j = 0; j <= numMismatchesToDisplay; j++) {
                    thisBase =
GetNthMostCommonBaseFromPileupString(splitArr[4], j + 1,
                        splitArr[2], ignoreGaps);
                    accurateSum =
GetNumberOfAccurateCallsInPileupString(splitArr[4],
                        splitArr[2], thisBase, ignoreGaps);
                    posArr[6 + (j * 2), thisPos] = thisBase;
                    posArr[6 + (j * 2) + 1, thisPos] =
                        Math.Round((double)accurateSum /
Convert.ToDouble(splitArr[3]), 3).ToString();
                }
                thisPos += 1;

            }
        }

    }
}

/// <summary>
/// Looks through the quality string, setting the "startingChar" value at 0
and difference from
/// subsequent quality score ASCII values to obtain an average quality score
for this position.
/// </summary>
/// <param name="qualStr">Pileup quality string for this position</param>
```

```
/// <param name="capAtK">Whether or not we set characters above "K" to "K"'s
score
///                     (to normalize Phred33+HiFi with Phred33)</param>
/// <param name="startingChar">Starting quality score character for this
run</param>
/// <returns>Double value with the average quality score at this
position</returns>
public double GetAverageQualityScoreFromString(string qualStr, bool capAtK =
true, char startingChar = '!') {
    int i;
    int startingVal = (int)startingChar;
    int maxVal = int.MaxValue;
    int thisVal;
    int charCount = 0;
    int valSum = 0;
    double avgScore;

    if (capAtK) {
        maxVal = (int)'K';
    }

    for (i = 0; i < qualStr.Length; i++) {
        thisVal = (int)qualStr[i] - startingVal;
        thisVal = Math.Min(thisVal, maxVal);

        charCount += 1;
        valSum += thisVal;
    }
    avgScore = (double)valSum / (double)charCount;
    return avgScore;
}

/// <summary>
/// Finds the Nth most common base in a SAMTools pileup string
/// </summary>
/// <param name="pileupStr">The SAMTools pileup string to examine</param>
/// <param name="positionRanking">The Nth position that we are trying to
examine (1 for most common,
///                               2 for 2nd, etc.)</param>
/// <param name="expectedBase">The base we expect to be aligned at this
position</param>
/// <param name="ignoreGaps">Whether or not to ignore gaps in the
alignment</param>
/// <returns>Returns a string with the Nth most commonly found
base.</returns>
public string GetNthMostCommonBaseFromPileupString(string pileupStr, int
positionRanking,
    string expectedBase = "", bool ignoreGaps = true) {
    pileupStr = GetNormalizedPileupString(pileupStr, true, ignoreGaps,
expectedBase);

    var charMap = pileupStr.Distinct().ToDictionary(c => c, c =>
pileupStr.Count(s => s == c));
```

```
    var baseList = charMap.OrderByDescending(kvp => kvp.Value).ToList();
    if (baseList.Count > positionRanking - 1) {
        return baseList[positionRanking - 1].Key.ToString();
    } else {
        return "";
    }
}

/// <summary>
/// Here we simply remove the base of interest from the pileup string and
then count of the difference
/// </summary>
/// <param name="pileupStr">The SAMTools pileup string to examine</param>
/// <param name="expectedBase">The base we expect to be aligned at this
position</param>
/// <param name="baseToFind">The base we are counting</param>
/// <param name="ignoreGaps">Whether or not to ignore gaps in the
alignment</param>
/// <returns>Returns an integer with the number of accurate calls for given
base</returns>
public int GetNumberOfAccurateCallsInPileupString(string pileupStr, string
expectedBase,
    string baseToFind, bool ignoreGaps) {
    string filteredStr;
    int accurateCalls = 0;

    if (!string.IsNullOrEmpty(baseToFind)) {
        pileupStr = GetNormalizedPileupString(pileupStr, true, ignoreGaps,
expectedBase);
        filteredStr = pileupStr.Replace(baseToFind, "");

        accurateCalls = pileupStr.Length - filteredStr.Length;
    }
    return accurateCalls;
}

/// <summary>
/// Normalizes a SAMTools pileup string for processing by downstream
functions:
/// </summary>
/// <param name="pileupStr">The SAMTools pileup string to be
processed</param>
/// <param name="allUppercase">Whether or not all bases should be converted
to upper case</param>
/// <param name="ignoreGaps">Whether or not to ignore gaps in
alignments</param>
/// <param name="expectedBase">The expected base in the pileup string, if
known</param>
/// <returns>Returns a string with a normalized pileup</returns>
string GetNormalizedPileupString(string pileupStr, bool allUppercase, bool
ignoreGaps,
    string expectedBase = "") {
        char[] newStr;
```

```
    string thisChar;
    string thisNum;
    int basesToDelete;

    if (!string.IsNullOrEmpty(expectedBase)) {
        pileupStr = pileupStr.Replace(".", expectedBase);
        pileupStr = pileupStr.Replace(",", expectedBase);
    }

    newStr = pileupStr.ToCharArray();

    for (int i = 0; i < newStr.Length; i++) {
        thisChar = newStr[i].ToString();
        //Mark special cases as "!", which will then be removed.
        if (thisChar == "^") {
            //Remove the caret plus any character afterwards, since it will
be a phred quality score
            //  for the read
            newStr[i] = Convert.ToChar("!");
            newStr[i + 1] = Convert.ToChar("!");
        } else if (thisChar == "+" || thisChar == "-") {
            //Leave the plus and minus, unless we choose to "IgnoreGaps"
            if (ignoreGaps) newStr[i] = '!';

            //remove the numbers and number of characters afterwards
            thisNum = "";
            for (int j = i + 1; j < newStr.Length; j++) {
                if (int.TryParse(newStr[j].ToString(), out _)) {
                    thisNum += newStr[j].ToString();
                    newStr[j] = Convert.ToChar("!");
                } else {
                    break; //exit for
                }
            }
            if (int.TryParse(thisNum, out _)) {
                basesToDelete = Convert.ToInt32(thisNum);
                for (int j = 0; j < basesToDelete; j++) {
                    //if (thisChar == "-" || j > 0) {
                    //backwards logic here, but we are avoiding the deletion
of the first character
                    // after the "+", since a "+2TA" is really an insertion
of an A after the T.
                    newStr[i + thisNum.Length + 1 + j] = Convert.ToChar("!");
                    //}
                }
            }
            if (thisChar == "-") {
                //Now we remove the "-" symbol, as these are shown as
astericks in the subsequent reads.
                newStr[i] = Convert.ToChar("!");
            }
        }
    }
```

```
    pileupStr = new string(newStr);
    pileupStr = pileupStr.Replace("$", "");
    pileupStr = pileupStr.Replace("!", "");
    pileupStr = pileupStr.Replace("^", "");
    pileupStr = pileupStr.Replace("]", "");
    pileupStr = pileupStr.Replace("*", "-");
    for (int i = 0; i <= 9; i++) {
        pileupStr = pileupStr.Replace(i.ToString(), "");
    }
    if (allUppercase) {
        pileupStr = pileupStr.ToUpper();
    }
    return pileupStr;
}

/// <summary>
/// Function to resize any type of 2D array to a new set of dimensions
/// </summary>
/// <typeparam name="T">Generic object type</typeparam>
/// <param name="original">Array to be resized</param>
/// <param name="x">New X dimension for zero-based array (array[X, Y]
format)</param>
/// <param name="y">New Y dimension for zero-based array (array[X, Y]
format)</param>
public void ResizeArray2D<T>(ref T[,] original, int x, int y) {
    T[,] newArray = new T[x, y];

    if (original != null) {
        int minX = Math.Min(original.GetLength(0), newArray.GetLength(0));
        int minY = Math.Min(original.GetLength(1), newArray.GetLength(1));

        for (int i = 0; i < minY; ++i)
            Array.Copy(original, i * original.GetLength(0), newArray, i *
newArray.GetLength(0), minX);
    }
    original = newArray;
}
```

References:

1. Cole, M.F. & Gaucher, E.A. Exploiting models of molecular evolution to efficiently direct protein engineering. *J. Mol. Evol.* **72**, 193-203 (2011).
2. Cacan, E., Kratzer, J.T., Cole, M.F. & Gaucher, E.A. Interchanging functionality among homologous elongation factors using signatures of heterotachy. *J. Mol. Evol.* **76**, 4-12 (2013).
3. Chen, F. et al. Reconstructed evolutionary adaptive paths give polymerases accepting reversible terminators for sequencing and SNP detection. *Proc. Natl Acad. Sci. USA* **107**, 1948-1953 (2010).
4. Gaucher, E.A., Miyamoto, M.M. & Benner, S.A. Function–structure analysis of proteins using covarion-based evolutionary approaches: elongation factors. *Proc. Natl Acad. Sci. USA* **98**, 548-552 (2001).
5. Smith, J.M. Natural selection and concept of a protein space. *Nature* **225**, 563-564 (1970).
6. Laos, R., Shaw, R., Leal, N.A., Gaucher, E. & Benner, S. Directed Evolution of Polymerases To Accept Nucleotides with Nonstandard Hydrogen Bond Patterns. *Biochemistry* **52**, 5288-5294 (2013).
7. Singh, I., Laos, R., Hoshika, S., Benner, S.A. & Georgiadis, M.M. Snapshots of an evolved DNA polymerase pre-and post-incorporation of an unnatural nucleotide. *Nucleic Acids Res.* **46**, 7977-7988 (2018).
8. Aye, S.L., Fujiwara, K., Ueki, A. & Doi, N. Engineering of DNA polymerase I from Thermus thermophilus using compartmentalized self-replication. *Biochem Biophys Res Commun* **499**, 170-176 (2018).
9. Lin, W., Zhang, X. & Seela, F. 7-Iodo-5-aza-7-deazaguanine: Syntheses of Anomeric D- and L-Configured 2-Deoxyribonucleosides. *Helv. Chim. Acta* **87**, 2235-2244 (2004).
