# Supplementary material for: Enzyme-assisted high throughput sequencing of an expanded genetic alphabet at single base resolution
Source: Nat Commun. 2024 May 14;15:4057. doi: 10.1038/s41467-024-48408-9 (PMC11094070; doi:10.1038/s41467-024-48408-9)
Supplement: Supplementary file 3 — Reporting Summary [file 41467_2024_48408_MOESM3_ESM.pdf]

nature portfolio

Corresponding author(s): Steven A. Benner
Last updated by author(s): Apr 7, 2024

Reporting Summary

Nature Portfolio wishes to improve the reproducibility of the work that we publish. This form provides structure for consistency and transparency in reporting. For further information on Nature Portfolio policies, see our Editorial Policies and the Editorial Policy Checklist.

Statistics

For all statistical analyses, confirm that the following items are present in the figure legend, table legend, main text, or Methods section.

n/a	Confirmed	
☐	☒	The exact sample size (*n*) for each experimental group/condition, given as a discrete number and unit of measurement
☐	☒	A statement on whether measurements were taken from distinct samples or whether the same sample was measured repeatedly
☒	☐	The statistical test(s) used AND whether they are one- or two-sided<br>*Only common tests should be described solely by name; describe more complex techniques in the Methods section.*
☒	☐	A description of all covariates tested
☒	☐	A description of any assumptions or corrections, such as tests of normality and adjustment for multiple comparisons
☐	☒	A full description of the statistical parameters including central tendency (e.g. means) or other basic estimates (e.g. regression coefficient) AND variation (e.g. standard deviation) or associated estimates of uncertainty (e.g. confidence intervals)
☒	☐	For null hypothesis testing, the test statistic (e.g. *F*, *t*, *r*) with confidence intervals, effect sizes, degrees of freedom and *P* value noted<br>*Give P values as exact values whenever suitable.*
☒	☐	For Bayesian analysis, information on the choice of priors and Markov chain Monte Carlo settings
☒	☐	For hierarchical and complex designs, identification of the appropriate level for tests and full reporting of outcomes
☒	☐	Estimates of effect sizes (e.g. Cohen's *d*, Pearson's *r*), indicating how they were calculated

Our web collection on statistics for biologists contains articles on many of the points above.

Software and code

Policy information about availability of computer code

Data collection	All chemical structures were drafted using ChemDraw software version 19.0. NMR spectra were recorded using a Varian Mercury M300 (Mercury300) spectrometer.
Data analysis	The bioinformatics analysis of the NGS data used standard software tools, such as FASTAptamer (2.0), BOWTIE2, and SAMTools, along with custom in-house scripts. The custom source code is available in Supplementary Note 1, which could also find in Zenodo: https://zenodo.org/records/10903570.

For manuscripts utilizing custom algorithms or software that are central to the research but not yet described in published literature, software must be made available to editors and reviewers. We strongly encourage code deposition in a community repository (e.g. GitHub). See the Nature Portfolio guidelines for submitting code & software for further information.

Data

Policy information about availability of data

All manuscripts must include a data availability statement. This statement should provide the following information, where applicable:

- Accession codes, unique identifiers, or web links for publicly available datasets
- A description of any restrictions on data availability
- For clinical datasets or third party data, please ensure that the statement adheres to our policy

The Supporting Information includes experimental procedures, materials and methods data, all synthetic dPTP derivatives, analytical characterization, and details of all assays. Additionally, the original gel images and statistical source data for this study are available in the Source Data file and are included with this paper.

Research involving human participants, their data, or biological material

Policy information about studies with human participants or human data. See also policy information about sex, gender (identity/presentation), and sexual orientation and race, ethnicity and racism.

Reporting on sex and gender	Our study's research does not include human participants or samples.
Reporting on race, ethnicity, or other socially relevant groupings	Our study's research does not include human participants or samples.
Population characteristics	Our study's research does not include human participants or samples.
Recruitment	Our study's research does not include human participants or samples.
Ethics oversight	Our study's research does not include human participants or samples.

Note that full information on the approval of the study protocol must also be provided in the manuscript.

Field-specific reporting

Please select the one below that is the best fit for your research. If you are not sure, read the appropriate sections before making your selection.

☒ Life sciences ☐ Behavioural & social sciences ☐ Ecological, evolutionary & environmental sciences

For a reference copy of the document with all sections, see nature.com/documents/nr-reporting-summary-flat.pdf

Life sciences study design

All studies must disclose on these points even when the disclosure is negative.

Sample size	The PAGE running experiment was conducted three times independently, each time yield similar results.<br>The translation of efficiency of (ATCGZP) to (ATCG) under various PCR conditions was statistically analyzed by examining every base in the all template DNA sequences, excluding the sequences of the primer region. the bases number (n>8).<br>For statistic the Cq of various PCR, the experiment were replicated for 3 times (n=3).<br>The statistic the called ZZ (5), the statistic the number of Z base (n=3).<br>All the qPCR experiments were conducted three time independently. (n=3)
Data exclusions	No data exclusions
Replication	Every experiment was independently replicated at least third time, yielding similar outcomes.
Randomization	Samples were randomly assigned to different experimental groups.
Blinding	In this study, data collection methods such as gel imaging, PCR data, and NGS data were conducted blind to the experimental conditions. For other experiments, although we did not implement single-blind procedures for sample identities, the data were derived from objective quantitative methods, minimizing the potential for subjective bias.

Reporting for specific materials, systems and methods

We require information from authors about some types of materials, experimental systems and methods used in many studies. Here, indicate whether each material, system or method listed is relevant to your study. If you are not sure if a list item applies to your research, read the appropriate section before selecting a response.

Materials & experimental systems

n/a	Involved in the study
☒	☐ Antibodies
☒	☐ Eukaryotic cell lines
☒	☐ Palaeontology and archaeology
☒	☐ Animals and other organisms
☒	☐ Clinical data
☒	☐ Dual use research of concern
☒	☐ Plants

Methods

n/a	Involved in the study
☒	☐ ChIP-seq
☒	☐ Flow cytometry
☒	☐ MRI-based neuroimaging

Plants

Seed stocks	Our study's research does not include Plants.
Novel plant genotypes	Our study's research does not include Plants.
Authentication	Our study's research does not include Plants.
